# Using evolutionary constraint to define novel candidate driver genes in medulloblastoma

**DOI:** 10.1073/pnas.2300984120

**Published:** 2023-08-07

**Authors:** Ananya Roy, Sharadha Sakthikumar, Sergey V. Kozyrev, Jessika Nordin, Raphaela Pensch, Suvi Mäkeläinen, Mats Pettersson, Zoonomia Consortium, Elinor K. Karlsson, Kerstin Lindblad-Toh, Karin Forsberg-Nilsson

**Affiliations:** ^a^Department of Immunology, Genetics and Pathology, Science for Life Laboratory, Uppsala University, 751 85 Uppsala, Sweden; ^b^Department of Medical Biochemistry and Microbiology, Science for Life Laboratory, Uppsala University, 751 23 Uppsala, Sweden; ^c^Broad Institute, Cambridge, MA 02142; ^d^Program in Molecular Medicine, UMass Chan Medical School, Worcester, MA 01605; ^e^Program in Bioinformatics and Integrative Biology, UMass Chan Medical School, Worcester, MA 01605; ^f^Division of Cancer and Stem Cells, University of Nottingham Biodiscovery Institute, Nottingham NG72RD, United Kingdom

**Keywords:** cancer, evolutionary constraint, regulatory mutations, medulloblastoma, pilocytic astrocytoma

## Abstract

Current knowledge of cancer genomics remains biased against noncoding mutations. To systematically search for regulatory noncoding mutations, we assessed mutations in conserved positions in the genome under the assumption that these are more likely to be functional than mutations in positions with low conservation. To this end, we use whole-genome sequencing data from the International Cancer Genome Consortium and combined it with evolutionary constraint inferred from 240 mammals, to identify genes enriched in noncoding constraint mutations (NCCMs), mutations likely to be regulatory in nature. We compare medulloblastoma (MB), which is malignant, to pilocytic astrocytoma (PA), a primarily benign tumor, and find highly different NCCM frequencies between the two, in agreement with the fact that malignant cancers tend to have more mutations. In PA, a high NCCM frequency only affects the *BRAF* locus, which is the most commonly mutated gene in PA. In contrast, in MB, >500 genes have high levels of NCCMs. Intriguingly, several loci with NCCMs in MB are associated with different ages of onset, such as the *HOXB* cluster in young MB patients. In adult patients, NCCMs occurred in, e.g., the *WASF-2/AHDC1/FGR* locus. One of these NCCMs led to increased expression of the SRC kinase *FGR* and augmented responsiveness of MB cells to dasatinib, a SRC kinase inhibitor. Our analysis thus points to different molecular pathways in different patient groups. These newly identified putative candidate driver mutations may aid in patient stratification in MB and could be valuable for future selection of personalized treatment options.

## Significance

Identification of cancer driver genes is important for understanding the biology of cancer and to define treatment targets. So far, protein-coding mutations have been frequently studied but less is known about regulatory noncoding mutation. Here, we identify noncoding constraint mutations (NCCMs) that are strong predictors of novel candidate cancer driver genes, defined primarily by regulatory changes. We find NCCMs in different genes in children and adult patients. This may lead to better stratification of medulloblastoma by age and subgroup and may improve targeted treatment strategies.

Systematic and genome-wide interrogations of human cancers have enabled the characterization of their mutational landscapes and generated comprehensive pan-cancer multiomics resources, intersecting mutational profiles with gene expression data (1). Cancer driver gene discovery has focused chiefly on coding mutations, with less attention paid to mutations in the noncoding parts, which make up >98% of the genome, though several papers have addressed noncoding mutations at scale (2–5). Although most noncoding mutations are expected to be passenger events, mutations in regulatory regions need a more in-depth analysis of their contribution to disease initiation and progression (6). The Pan-Cancer Analysis of Whole Genomes consortium, investigating 2,658 tumors from 38 cancer diagnoses, highlighted the prevalence of *TERT* promoter mutations but reported a very limited number of new potential driver events in noncoding regions (7). Dietlein et al recently applied a genome-wide approach to define and analyze noncoding positions by looking for enrichment of mutations within genomic regions within and between tumor types (8).

The two most common pediatric brain tumors are pilocytic astrocytoma (PA) and medulloblastoma (MB). PA is relatively benign and has few mutations, with fusion events of the BRAF kinase domain with the N-terminus of KIAA1549 being the predominant genetic alteration. MB, on the other hand, is malignant and can be divided into four molecular subgroups; WNT (Wingless), SHH (Sonic Hedgehog), group 3, and group 4 (9). MB can also be found in adults albeit as a rare tumor (10). The WNT subgroup has the best prognosis, with > 90% survival in all age groups. This group carries germline mutations of *APC*, a WNT pathway inhibitor, as well as somatic mutations in *CTNNB1* and high protein expression of ß-catenin. The SHH subgroup patients have a bimodal age distribution into infants and adults, with adults having a higher mutational burden (9). Adult SHH patients have mutations in the SHH signaling pathways, including *PTCH*, *SMO*, and *SUFU*, as well as amplifications of *GL1* and *GL2*. Group 3 MB occurs in infants and children and has the worst clinical outcome (5-y overall survival <40%). Group 3 MB tumors are genetically characterized by *MYC* amplifications, with additional *MYC*, *PVT1*, *OTX2*, *MLL2*, *SMARCA4,* and *CHD7* mutations being reported. Group 4 MB occurs across all age groups. With an intermediate prognosis, group 4 MB accounts for almost 35% of the MB patients and is characterized by *MYCN* and *CDK6* amplifications. For both groups 3 and 4, >30% of the patients have metastatic disease at diagnosis.

A systematic way to identify which positions in the genome are important for function can be the study of evolutionary constraint (11). We hypothesize that a position under evolutionary constraint in the genome can be leveraged to identify functional sequences and deleterious mutations. In a proof-of-concept study, we previously used GERP scores, a widely used constraint metric, to discover noncoding constraint mutations (NCCMs) in glioblastoma (12). Recently, we identified single-base phyloP constraint scores from the whole-genome alignment of 240 placental mammals (13, 14) and found that 3.5% of bases in the human genome are significantly constrained (15). As the Zoonomia data predict that >10% of the genome is constrained, we decided to use 8% as an intermediate compromise for this study. Based on a comparison to published large-scale genome annotation, and other data sets, we proposed that evolutionarily constrained positions are enhanced for variants behind human disease (15). In the present study, we build on, and expand, our initial analysis. We here apply phyloP scores to whole-genome data of MB and PA to identify genes with increased noncoding constraint mutational burden and potential function in cancer.

## Results and Discussion

### Mutation Counts for Medulloblastoma Are Higher than What Is Observed for Pilocytic Astrocytoma.

We downloaded variant calls from whole-genome sequencing (WGS) data from the International Cancer Genome Consortium (ICGC) (1, 16) for pilocytic astrocytoma [PA, 89 cases, (Dataset S1) (17)] and medulloblastoma [MB, 146 cases (Dataset S2)] (16). Total mutational counts, when normalized by patient numbers, were significantly higher for MB than PA, reflecting their different grades (*SI Appendix*, Fig. S1). To ensure the accuracy of our analysis, we first confirmed that we detected the protein-coding mutations previously identified by the ICGC (18). To investigate mutations in the noncoding part of the genome for potential functional impact, somatic alterations in introns, 5′ and 3′ UTRs, as well as intergenic regions ±100 *kbp* upstream and downstream of each gene (excluding positions in protein-coding sequence), were analyzed for NCCMs. Previously, estimates have been made that between 6 and 13% of the human genome is functional (19–21). With the whole-genome alignment of 240 placental mammals (13), we identify 3.5% of the genome as significantly constrained. As the Zoonomia data predict that >10.7% of the genome is constraint, we choose an intermediate cutoff at 8% constrained positions, similar to that used by us in a prior study to examine NCCMs in glioblastoma (12). Based on single-base phyloP scores from the Zoonomia data (13), this corresponds to phyloP values ≥1.2. The noncoding variants in PA and MB that met the selection criteria for constraint, are hereafter referred to as NCCMs. We further use ≥2 NCCMs/100 *kbp,* empirically determined for our previous study (8), where we used 78 key GBM genes, as a basic cutoff to define candidates. We implement this as a rate per sample, to make the comparisons between studies with different sample sizes more equivalent.

### Mutations in PA Are Primarily Found in BRAF.

We first examined PA, where *BRAF* fusions are the main events (22). Our NCCM analysis showed only one gene, *NDUFB2* with ≥2 NCCMs/100 *kbp*. *NDUFB2* is located in the same locus as *BRAF* and *ADCK2*, both with ≥1 NCCMs/100 *kbp*, sharing these NCCMs (*SI Appendix*, Fig. S2 *A* and *B*). We found 4 NCCMs in genes previously reported to be significantly mutated in PA (*SI Appendix*, Fig. S2*C* and Dataset S3). Examining nine additional genes with ≥1 NCCMs/100 *kbp* (Dataset S4), we note the nutrient sensor *OGT*, a kinase that is overexpressed in PA (23). Significantly mutated genes that have alterations in the protein-coding sequence (Dataset S3) and the genes carrying ≥1 NCCMs/100 *kbp* (Dataset S4) were jointly subjected to gene set over-representation analysis, which revealed enrichment for ‘diseases of signal transduction’ (*P*-value = 1.8 × 10^−4^) and ‘oncogenic MAPK signaling’ (*P*-value = 1.7 × 10^−3^) (Dataset S5), supporting the relevance of NCCM enrichment for PA. We hypothesize that these mutations are kept even in the presence of the *BRAF* translocation, as they might alter expression of the *BRAF* gene and thereby nurture tumorigenicity. Thus, *BRAF* together with a few additional genes with NCCMs, point strongly to a mechanism related to previously identified alterations in signal transduction, with no additional noncoding driver genes found in this study for PA.

### Genes with NCCMs in MB Are Enriched for Nervous System Development.

In the MB cohort, 114 genes had a high NCCM occurrence rate, i.e., ≥ 2 NCCMs/100 *kbp* (*SI Appendix*, Fig. S3*A* and Datasets S6 and S7). To complement this weighted analysis, we also performed a simplified analysis identifying 525 genes with ≥ 5 NCCMs (*SI Appendix*, Fig. S3*B* and Dataset S8). These sets together (n = 530, See *SI Appendix*, Fig. S3*C*) were enriched for ‘nervous system development’ (*P*-value = 1.3 × 10^−26^) and ‘generation of neurons’ (*P*-value = 1.7 × 10^−22^), as shown by gene set analysis (Dataset S9). We also observed a moderate correlation between the number of NCCMs and the gene length (*SI Appendix*, Fig. S4*A*), which suggests that the analysis of all genes with ≥ 5 NCCMs slightly favors longer genes, and we, therefore, focused our analysis on the genes with ≥ 2 NCCMs/100 *kbp*. In the majority (81%) of these 114 genes, NCCMs were evenly distributed across patients, with one NCCM per patient. In 19% of the 114 genes with the highest NCCM rates, up to two NCCMs were found in the same patient (Dataset S7). There were few coding mutations in the top 114 genes (Dataset S10), and the genes with protein-coding mutations had low NCCM rates (Dataset S11). Thus, NCCMs and coding mutations generally do not affect the same set of genes, and our analysis reveals a large number of new mutations, and new genes of interest.

In line with the total mutational burden being higher for MB than for PA, cohort-wide normalization of NCCMs showed that MB had greater NCCM accumulation rates than PA. This was the case both when comparing all genes (*SI Appendix*, Fig. S4*B*) and the top 0.5% of genes with NCCMs, in each cohort (*SI Appendix*, Fig. S4*C*). To determine whether our increased NCCM rates in specific genes were a function of potential confounders such as regional mutation rates (*SI Appendix*, Fig. S4*D*), high GC-content (*SI Appendix*, Fig. S4*E*), or low-complexity regions (*SI Appendix*, Fig. S4*F*), we analyzed the correlation of these confounding factors with the NCCM rates of the genes. We found no correlations (*SI Appendix*, Fig. S4 *D*–*F*) between them, suggesting that the enrichment of NCCMs is a factor of evolutionary selection for functionality, rather than an effect of the confounders. Comparing DNase I hypersensitive annotations as a function of accessible genome position, we observe >45% of our NCCMs overlay with the active regions and >20% with transcriptionally active chromatin mark (H3K4me3), thus indicating their potential epigenetic regulatory role and clinical potential as patient stratifiers (Dataset S7).

### NCCMs and Coding Mutations Show a Differential Distribution Pattern.

Analysis of single-base substitution pattern in the MB patients displayed a differential distribution pattern of the mutational signatures between coding, noncoding constraint, and noncoding nonconstraint mutations. Of the total mutation counts, the variants that were assigned to signatures were an average of 80 NCCMs, 1,349 noncoding nonconstraint mutations, and 14 coding mutations per sample. Most of the signatures, i.e., SBS1, SBS5, SBS8, and SBS18, that we find relate to what has previously been reported for pediatric cancers (24). Our analysis also shows traces of signatures related to defective DNA base repair SBS30 as well as SBS39 and SBS40 (25, 26). An interesting observation was the shift of the clock-like signatures between the coding and NCCM mutations. NCCMs had a significantly lower level of the SBS1 signature (Fisher’s exact test, *P*-value = 1.7 × 10^−159^), whereas an increased proportion of SBS5 was seen in NCCMs (Fisher’s exact test, *P*-value = 8.3 × 10^−22^), compared to the coding variants (*SI Appendix*, Fig. S5*A*).

### Genes with NCCMs Are Differently Distributed across Pediatric and Adult MB.

**Fig. 1.**
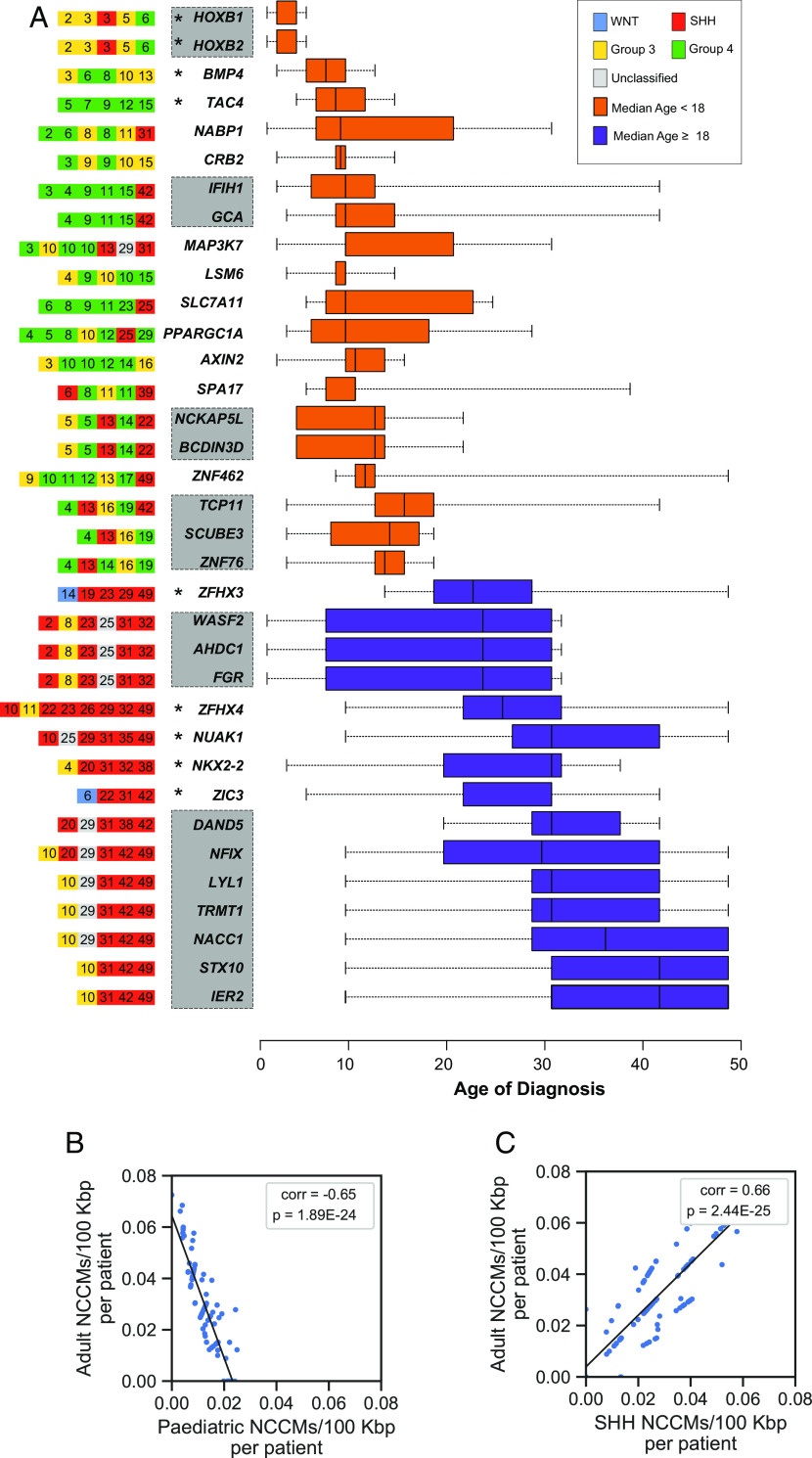
Candidate driver genes for MB have ≥ 2 NCCMs per 100 kbp and are differently distributed across pediatric and adult MB. (*A*, *Left*) subgroup (by color code), and age (denoted by the number in the squares) for each patient. (*A*, *Right*) Age of onset distribution. Orange, median age <18, purple, median age >18. Gray boxes denote genes in the same locus (See *SI Appendix*, Fig. S5 for all 114 genes). Asterisks denote genes reported by us in ref (15). (*B*) Negative correlation in NCCMs/100 kbp per patient between adult and pediatric patients. (*C*) Positive correlation between NCCMs/100 kbp per patient in adult and SHH group.

Querying the number of NCCMs in known MB driver pathways revealed 29 NCCMs in nine genes of the SHH pathway (of which nine were in SHH group patients), 28 NCCMs in 10 out of 12 genes of the WNT pathway, and 22 NCCMs in 10 genes in the MYC pathway (Dataset S12). Many of these mutations were distributed across the different subgroups, showing a considerable overlap of pathways. SHH tumors had the highest overall count of these NCCMs, but in group 4 the rate per patient was highest (*SI Appendix*, Fig. S5 *B* and *C*). We also note a demarcation between NCCM-containing genes occurring predominantly in children (<18 y) or adults (≥18 y) (*SI Appendix*, Fig. S6). There are distinct early and late-onset genes suggesting different driver genes in the different age groups [Fig. 1*A* and (15)]. There is a highly significant negative correlation between adult and pediatric NCCMs/100 *kbp* per patient (Fig. 1*B*) strengthening the distinction of different effector pathways in the age groups. In addition, we find a positive correlation when comparing adult patient NCCMs and those in the SHH group (predominant subgroup in adult patients) (Fig. 1*C* and *SI Appendix*, Fig. S7*D*). Similar to the NCCM correlation, we observed a weak negative association of the coding mutation rates while comparing the pediatric to the adult group (*SI Appendix*, Fig. S7*K*).

The vast majority, 81%, of pediatric MB patients had at least one NCCM, and 39% had NCCMs in more than one locus (*SI Appendix*, Fig. S8). In the pediatric cohort, most genes with NCCMs are associated with group 3 and group 4 (Fig. 1). These subgroups remain difficult to distinguish because few group-specific recurrent protein-coding mutations have been reported (16, 27). We, therefore, examined the NCCM-containing genes for possible preponderance toward either of the subgroups. *BMP4, CRB2, LSM6,* and *AXIN2* had NCCMs exclusively in group 3 and 4 patients, while no NCCMs were private to group 3. *TAC4* was exclusive to group 4, and the loci for *IFIH1/GCA* and *SLC7A11* were exclusive to group 4 and one SHH patient. *SLC7A11* is linked to seizures in glioma (28), and intriguingly, four of the *SLC7A11* NCCMs are shared with its corresponding *SLC7A11* antisense transcript, showing regulatory potential. Because 24% of group 3 and 18% of group 4 cases lack potential coding driver mutations (16), the above NCCM information could identify new candidates for these subgroups.

Mutations in the promoter region of the telomerase reverse transcriptase (*TERT*) gene is a common recurrent somatic noncoding mutation in MB (29) with predominant enrichment for SHH and WNT groups in adult patients. In the current cohort, 5 out of the 37 patients (14%), all belonging to the SHH group, have mutations at the chr5:1,295,228 position but none at the chr5:1,295,250 position. No constraint positions close to the *TERT* promoter region were found to be mutated in this dataset. *TERT* promoter mutations are well-established noncoding drivers in MB (16) but the *TERT* promoter mutations for the analyzed dataset fell below the constraint cutoff criteria of phyloP values ≥1.2 used in the present study.

### The BMP4 Gene Carries NCCMs that May Alter Transcription Factor Binding.

**Fig. 2.**
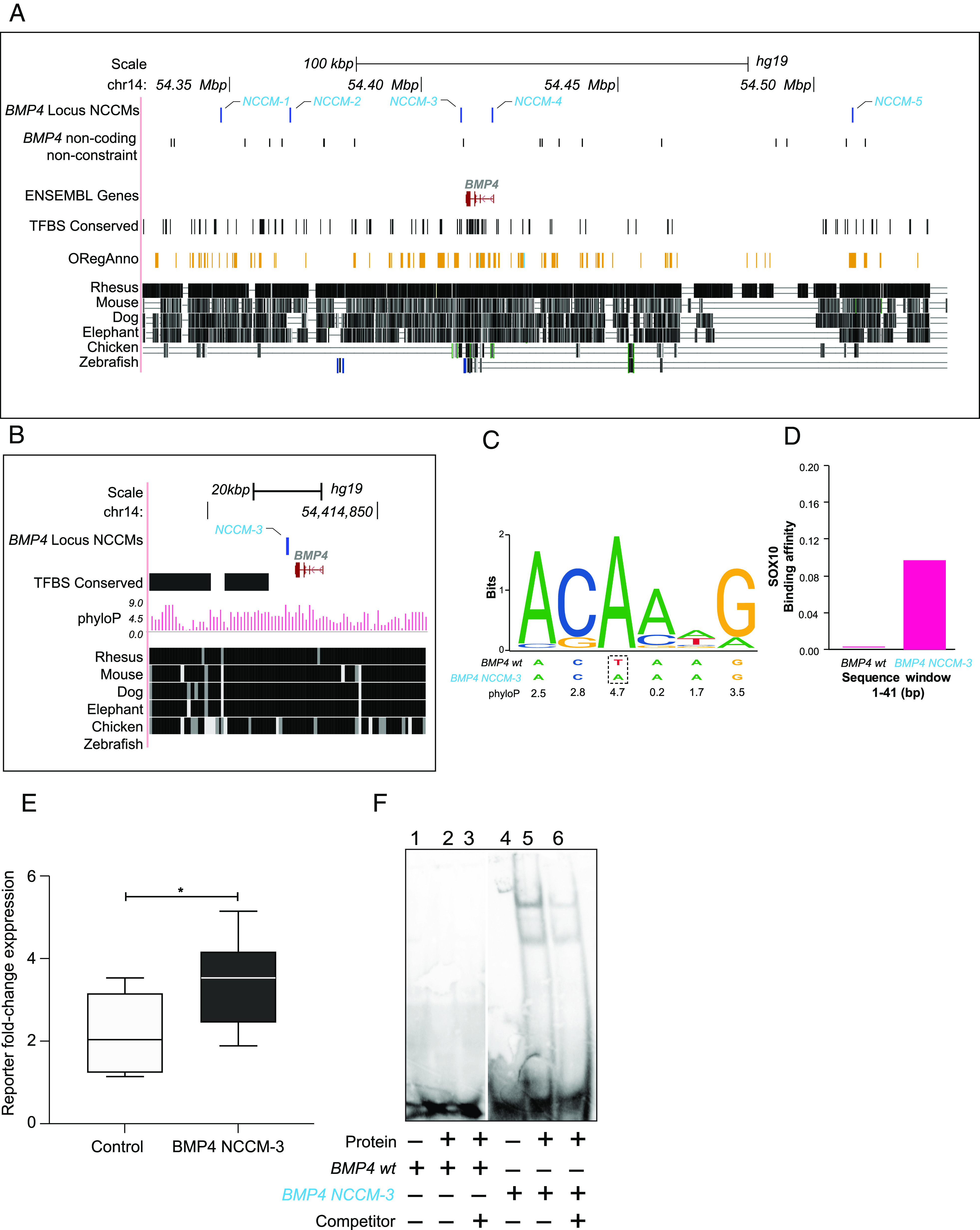
*BMP4*, a possible candidate gene for young MB patients. (*A*) The five *BMP4*-NCCMs (blue) all lie in regions of high constraint and have at least one regulatory annotation. NCCMs and noncoding nonconstraint mutations are shown on individual rows. (*B*) Zoomed-in view of BMP4-NCCM-3 shows high phyloP score. (*C*) SOX10 binding logo shows *BMP4*-NCCM-3 in a highly conserved position. (*D*) Bioinformatic prediction is that SOX10 affinity increases for the mutated versus wild-type sequence. (*E*) Reporter assay shows significantly increased expression of the reporter gene for the mutant allele for NCCM-3 compared to the wild type in MB002 cells. (*F*) EMSA with nuclear protein extract from MB002 cells shows increased binding to the mutant (lanes 5 to 6) compared to wild-type sequence (lanes 2 to 3). Unlabeled dsDNA was used as competitor (lanes 3 and 6). Unpaired Student *t* test was used to analyze significance. * indicates *P* ≤ 0.05.

Among the youngest patients, we find NCCMs in the loci for *BMP4* and the *HOXB* gene family [Fig. 1*A* and (15)]. BMP4 is a pivotal regulator of neural development that induces differentiation of cerebellar progenitors (30) and has been proposed to suppress MB in mice (31). Of five *BMP4* NCCMs (Fig. 2*A*), we selected NCCM-3 (Fig. 2*B*) for functional studies because this position shows high conservation in multiple transcription factor binding sites, e.g., SOX10 (Fig. 2*C*) and because sTRAP analysis predicted increased affinity of SOX10 binding, compared to the wild-type sequence (Fig. 2*D*). A reporter assay showed increased activity of the region (Fig. 2*E*), and electrophoretic mobility shift assay (EMSA) using nuclear extracts from the MB cell line MB002 (Fig. 2*F*) confirmed the prediction that NCCM-3 increases DNA binding in this region. Interestingly, NCCMs in the *TCP11/SCUBE3/ZNF76* locus (Fig. 1*A*) support the notion of the BMP4 pathway as a potential MB candidate mechanism because SCUBE3, a glycoprotein anchored to the cell surface, is a coreceptor for BMP2/BMP4 (32). However, more studies of the *BMP4* NCCMs are needed to verify what functional consequences these may have for tumor growth. Notably, data from Taylor et al. (33) and Mackay et al. (34) support the notion of age differences in mutations of the BMP pathway for malignant brain tumors, as they reported activating mutations in BMP receptor *ACVR1* in the youngest fraction of diffuse intrinsic pontine glioma patients.

### The HOXB Gene Cluster Contains Highly Constraint NCCMs that Alter Gene Expression.

**Fig. 3.**
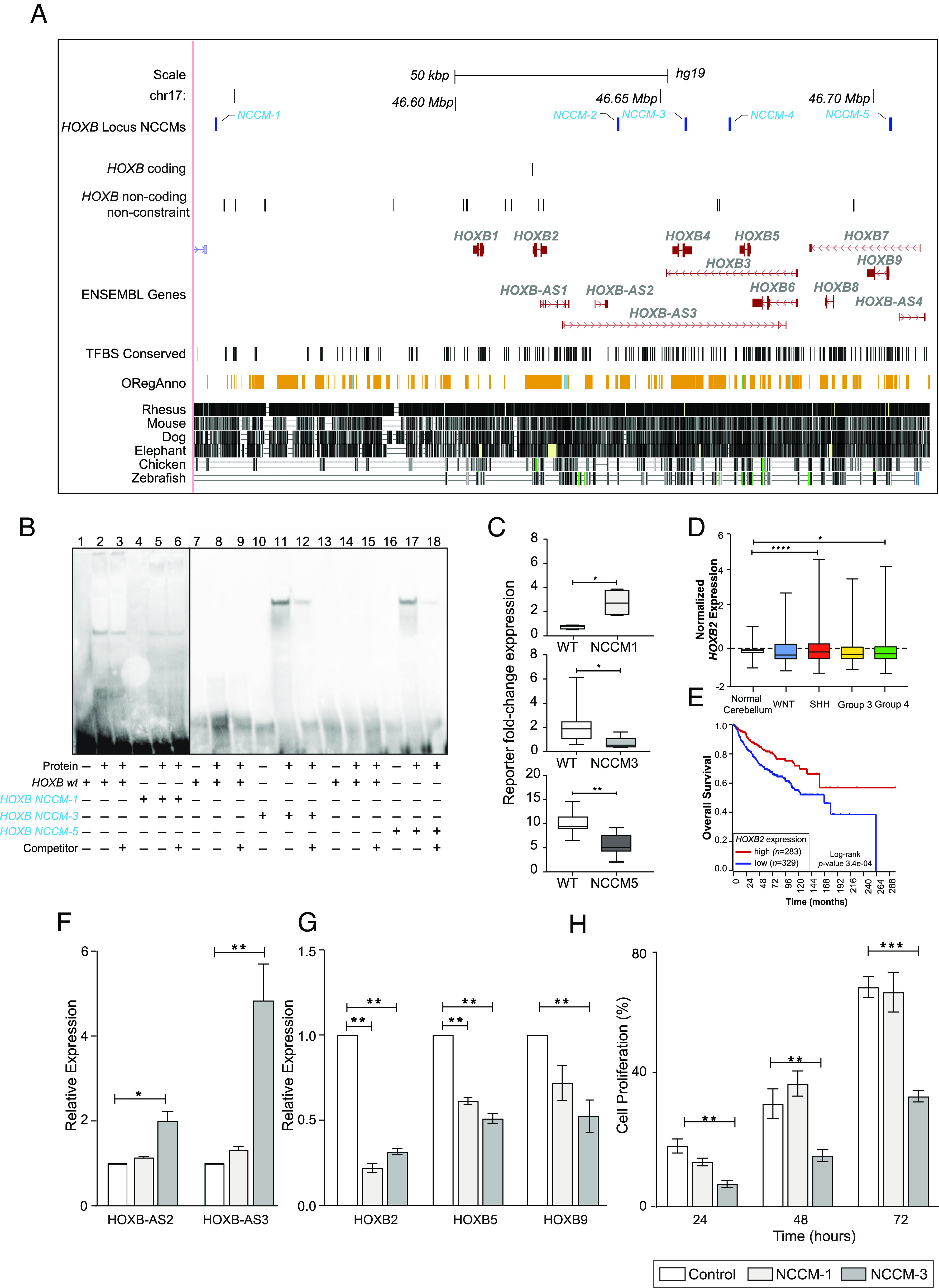
The *HOXB* gene cluster contains five highly constraint NCCMs. (*A*) Five NCCMs (blue) are found in the *HOXB* locus, which contains nine protein-coding genes and multiple antisense transcripts. These NCCMs are highly constraint and have at least one regulatory annotation each. (*B*) EMSAs with nuclear protein extracts from MB002 cells show slightly decreased binding for NCCM-1 (lanes 5 to 6) compared to wild type (lane 2 to 3). In contrast, an increased binding to HOXB-NCCM-3 (lanes 11 to 12) and HOXB-NCCM-5 (lanes 17 to 18), compared to wild-type sequences (lanes 8 to 9 and 14 to 15, respectively) is seen. Unlabeled dsDNA was used as competitor (lanes 3, 6, 9, 12, 15, and 18). (*C*) Reporter assay shows increased expression of the mutant allele for NCCM-1 (*Upper*), and decreased expression of the mutant alleles for NCCM-3 (*Middle*) and NCCM-5 (*Lower*). (*D*) Expression of *HOXB2* in normal cerebellum and MB patients. Boxes min–max range, horizontal lines median value, and whiskers extend to extreme values. (*E*) Kaplan–Meier plot showing that patients with lower *HOXB2* expression have shorter survival than those with high expression. (*F*) Upon CRISPR/Cas9-editing, the expression of *HOXB* antisense transcript 2 (*HOXBAS2*) and *HOXBAS3* is increased as a consequence of inserting NCCM-3 in DAOY cells. The NCCM-1 did not show such an effect. (*G*) DAOY cells edited to contain NCCM-1 or NCCM-3 show reduced expression of *HOXB2* and *HOXB5*, and NCCM-3 also reduced *HOXB9* expression. (*H*) Proliferation assay comparing CRISPR/Cas9 cell edits for NCCM-1 or NCCM-3 to control-edited DAOY cells, showing reduced proliferation by NCCM-3 but not by NCCM-1. Unpaired Student *t* test with Welch’s correction was used to analyze significance. *, **, *** indicates *P* ≤ 0.05, *P* ≤ 0.01 and *P* ≤ 0.001, respectively.

HOXB proteins are instrumental in hindbrain development (35), where they spatially restrict neural progenitors to ensure segmental ordering of the rhombomeres. Five patients had NCCMs in the *HOXB* locus (Fig. 3*A*), which also contains several *HOXB*-antisense transcripts. The sTRAP analysis predicted that *HOXB-*NCCM-3 increases the binding affinity of FOXD1, FOXD3, and FOXF2, and *HOXB-*NCCM-5 affects the PAX6 and POU5F1/OCT4 binding (Dataset S13). We confirmed differential binding to the regions of NCCM-1, NCCM-3, and NCCM-5 using EMSAs (Fig. 3*B*). Altered reporter transcript expression for the mutant alleles of NCCM-1, NCCM-3, and NCCM-5 were also established (Fig. 3*C*). Of the *HOXB* locus genes, *HOXB2* stands out since SHH and group 4 patients have lower *HOXB2* expression compared to normal cerebellum (36) (Fig. 3*D*), and patients with low *HOXB2* expression have shorter survival (*P* = 3.4 × 10^−4^) (Fig. 3*E*). Comparing DNase I hypersensitivity as an indication of chromatin accessibility, the *HOXB* NCCMs reside in accessible regions proposing that these positions are functional. Examining *HOXB2* in the literature, we noted that DDX3X has tumor suppressor capacity in the hindbrain by regulating the expression of, e.g., *Hoxb2* (37). *DDX3X* is frequently mutated in WNT and SHH MB (37), which supports the importance of *HOXB* mutations in MB, but until now, it has not been described for group 3 and group 4 patients. To further characterize the NCCM effects on the *HOXB* locus, we generated CRISPR/Cas9-edited MB cells (DAOY) and found that the introduction of NCCM-3, but not NCCM-1, significantly increased the expression of antisense transcripts *HOXB-AS2* and *HOXB-AS3* (Fig. 3*F*). In addition, the introduction of NCCM-1 and NCCM-3 both resulted in reduced expression of *HOXB2* and *HOXB5,* and NCCM-3 also reduced *HOXB9* expression (Fig. 3*G*). Proliferation assays reflect a lower proliferation rate of DAOY cells with NCCM-3, whereas for NCCM-1, it was comparable to control, non-edited, cells (Fig. 3*H*). Thus, a complex regulatory pattern for the *HOXB* locus with multiple genes affected by NCCMs was demonstrated, including alterations in MB cell proliferation.

### Adult-Onset Medulloblastoma Shows Distinct NCCMs.

Most NCCMs associated with adult-onset were found in genes normally expressed in the brain, many with relatively high expression in cerebellum: *ZFHX4, WASF2/AHDC1/FGR, NUAK, ZIC3*, *DAND5/NFIX*/*STX10* (https://www.gtexportal.org/home/). There were NCCMs in at least one of these loci (*SI Appendix*, Fig. S9) in 50% of SHH patients. *ZFHX4*, with nine NCCMs in seven patients, either in introns or upstream of the gene, and overlapping a *ZFHX4-antisense* transcript (15), has been reported to be down-regulated in MB (38). *ZFHX3,* on the other hand, is normally not expressed in the brain but has been associated with multiple cancers where it acts as a tumor suppressor by down-regulating MYC (39). Cancers with high MYC levels depend on the Ser/Thr kinase, NUAK1, to promote spliceosome activity, revealing a MYC-sensitive feedback control of transcription (40). This is intriguing, as *NUAK1* has six NCCMs in five patients (Dataset S7) and suggests that NUAK1 inhibitors (31) may be tested for efficacy on MB models with high *MYC* or *NUAK1* expression and/or low *ZFHX3* expression.

### NCCMs in the NFIX Locus of Adult Medulloblastoma Alter the Expression of Several Genes.

Six NCCMs were found in the *NFIX* locus (*SI Appendix*, Fig. S10*A*) with seven genes showing expression in the brain, specifically the cerebellum (https://www.gtexportal.org/home/). These fall within the same topologically associated domain (TAD), suggesting potential coregulation (*SI Appendix*, Fig. S10*B*), and published datasets showed that *NFIX* expression is higher in SHH MB compared to normal cerebellum (*SI Appendix*, Fig. S10*C*) (36). Furthermore, *DAND5*, a secreted BMP antagonist (41) in the same locus, could be tumor-promoting by maintaining low BMP levels. Recreating NCCM-2 and NCCM-4 of this locus in DAOY MB cells with CRISPR/Cas knock-in editing show differences in expression for multiple genes compared to the nonedited control cells. *NFIX* is down-regulated by NCCM-2, while *NACC1* shows increased expression by NCCM-4*. LYL1* was up-regulated both by NCCM-2 and NCCM-4 while *TRMT1* was unchanged (*SI Appendix*, Fig. S10*D*). While reporter assays and proliferation patterns for the edited variants did not reach significance (*SI Appendix*, Fig. S10 *E*–*G*), several oncogenic roles have been ascribed to genes of this locus, which merits further investigations of possible phenotypes associated with these NCCMs.

### NCCMs in the WASF2/AHDC1/FGR Locus Alter the Drug Response of Medulloblastoma Cells.

**Fig. 4.**
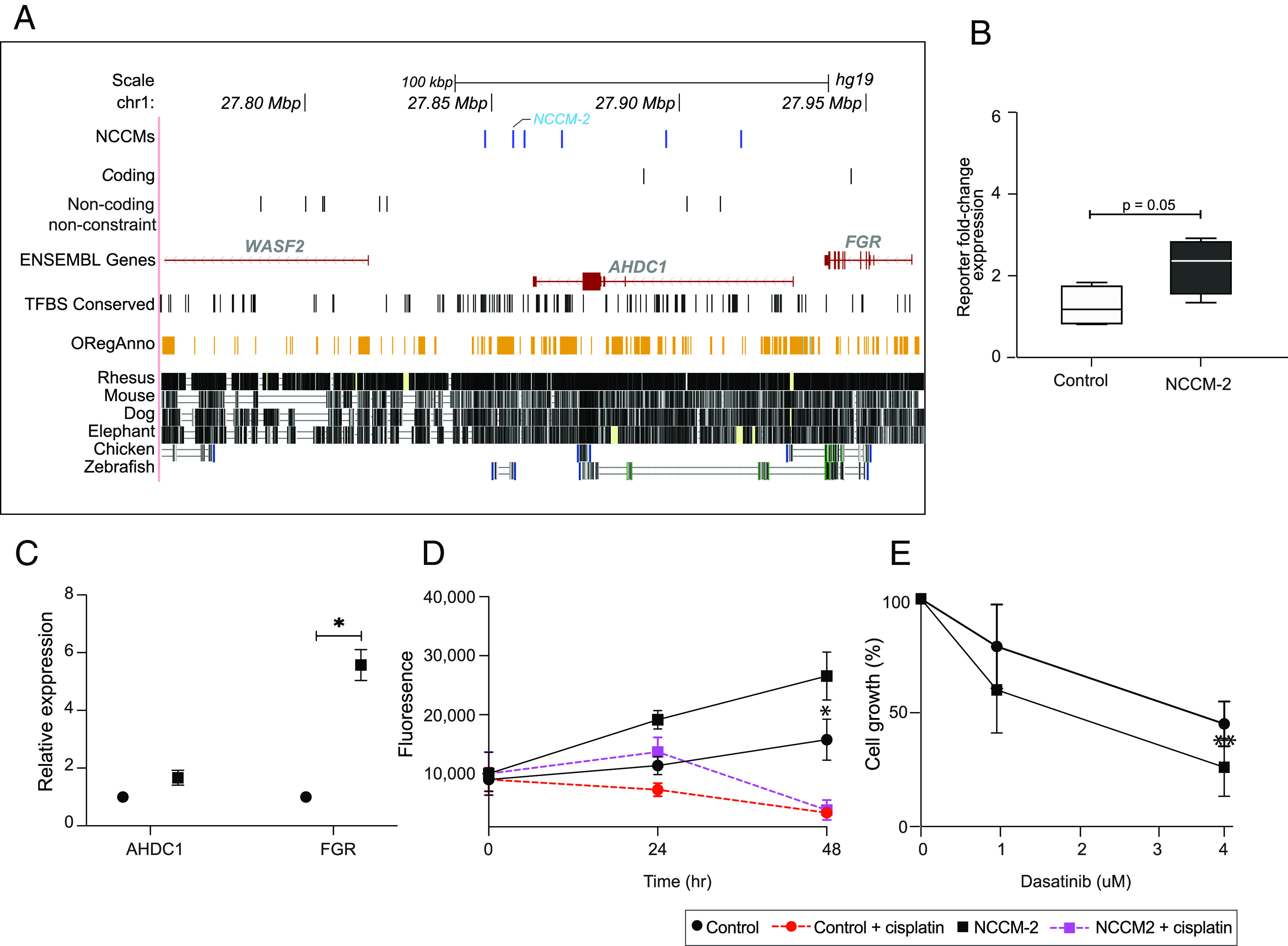
NCCMs in the *ADHC1WASF2,* and *FGR* genes mainly in adult patients. (*A*) Six NCCMs (blue), with regulatory potential in the introns of the *ADHC1* gene are close to *WASF2* and *FGR* and found in areas of high mammalian conservation. (*B*) Allelic effects of the *AHDC1* NCCM-2 on reporter transcript expression shows near significant (*P* = 0.05) fold change relative to the level of the wild-type reporter vector in the MB002 cells. (*C*) CRISPR/Cas9 editing of NCCM-2 in DAOY cells leads to increased expression of *FGR*. (*D*) DAOY cells with NCCM-2 show faster growth than control DAOY cells, and both are growth-inhibited by cisplatin (1 μg/mL) treatment. (*E*) Dasatinib, a SRC kinase inhibitor, more efficiently decrease the growth of the NCCM-2 CRISPR/Cas9 DAOY cells, compared to control cells after exposure for 72 h. Unpaired Student *t* test with Welch’s correction was used to analyze significance. * indicates *P* ≤ 0.05.

The locus containing *WASF2/AHDC1/FGR,* with six NCCMs (Fig. 4*A*), is highly conserved, and microdeletions/duplications involving *AHDC1* have been linked to neurodevelopmental disorders (42). A reporter assay showed that the NCCM-2 mutation increased reporter expression close to significant levels (*P* = 0.05, Fig. 4*B*) in the MB cell line MB002. To investigate the functional potential of NCCM-2 in this locus, we used CRISPR/Cas9 to insert NCCM-2 in DAOY cells. While expression of *AHDC1* did not markedly change, expression of the neighboring gene, *FGR*, which shares the same TAD (*SI Appendix*, Fig. S11*A*), increased upon gene editing (*P* = 0.02, Fig. 4*C*). *FGR* is a nonreceptor tyrosine-protein kinase of the SRC family, with oncogenic capacity in AML (43) but not previously implicated in MB. DAOY cells with NCCM-2 showed a significantly increased proliferation rate (Fig. 4*D*) compared to control cells. Both CRISPR/Cas9-edited and control DAOY cells were growth-arrested by cisplatin, a chemotherapeutic agent used widely as treatment of MB (44, 45), while dasatinib, a SRC family kinase inhibitor (46), reduced the proliferation of NCCM-2-edited DAOY cells more efficiently than controls (Fig. 4*E*). This suggests that NCCMs could alter drug response of MB cell, which is further supported by the observation by Wilson et al. (47) that FGR1 is part of the resistance mechanism to ALK inhibitors in lung cancer.

In conclusion, using phyloP constraint scores from 240 mammals, we have shown that genes with NCCMs are associated with different ages of onset and MB subgroups. Several novel candidate driver genes have plausible roles in MB development, and NCCMs could therefore be of diagnostic and/or of therapeutic potential.

## Materials and Methods

### Cohort Information and Data Access.

All WGS data used in this project are a part of the International Cancer Genome Consortium (ICGC) project PBCA-DE. Access to the controlled data set for analysis in this project was granted to the authors by the ICGC Data Access Compliance Office. Somatic mutation files in variant calling format corresponding to 89 pilocytic astrocytoma (PA) and 146 medulloblastoma (MB) patient samples were downloaded from the ICGC data portal (https://dcc.icgc.org/releases/PCAWG/consensus_snv_indel/) (16, 17, 48). The raw sequence data corresponding to these analysis-ready files was aligned to the human genome build hg19. Consequently, all downstream analyses performed on the data use this version of the human genome. The cohort comprised 47 females and 41 males for the PA set, and the median age of diagnosis was 8 y (range: 1 to 50). Similarly, among the MB cohort of 66 females and 90 males, the median age of diagnosis was 9 y (range: 1 to 49). The molecular subgroups for the MB tumors include WNT (*n* = 3), SHH (*n* = 37), group 3 (*n* = 43), and group 4 (*n* = 60). For 3 MB samples, there was no information available for the molecular histological classification.

### Classification of Variant Calls and Identification of Significantly Mutated Genes Based on Protein-Coding Mutations.

GATK’s Funcotator module (GATK 4.1.4.) was used to functionally classify the MB and PA somatic point mutation (SPM) and somatic indel mutations (SIM) as being either coding or noncoding changes. The coding changes were subsequently input to the algorithm MutSigCV (v1.41) to identify significantly mutated genes, genes with a statistically higher rate of somatic changes than expected by random chance.

### Identification of NCCMs.

All of the SPM and SIM calls were annotated with phyloP scores to identify NCCMs. The human genome hg38-phyloP scores (a measure of evolutionary constraint based on the alignment of 240 mammals) were first converted to the hg19 genome using the UCSC genome browser utility ‘liftOver’, after which the annotation was applied to the variant calls. The phyloP scores range from a minimum of −20 to a maximum of +9, corresponding to regions of accelerated evolution to highly conserved regions in the genome. Prior estimates of the fraction of the human genome that is functional have been between 6 and 13%. Hence, we used an intermediate estimate of the top 8% constraint positions previously used by us to examine NCCMs in glioblastoma (12). This corresponds to phylo *P* values ≥ 1.2. The noncoding variants that met the selection criteria for constraint, hereafter referred to as NCCMs, were then chosen for additional study. First, NCCMs located within 3′- or 5′-UTRs, introns, or ±100 *kbp* intergenic flanking regions of protein-coding genes were extracted. Next, the rate of NCCMs, normalized to the length of the queried genomic region around each gene was computed, and those genes that had ≥2 NCCMs per 100 *kbp* were delineated as candidate driver genes (*n =* 114 for MB). In addition, we identified all genes in MB with ≥5 NCCMs in the target area (±100 *kbp*) for further analysis (*n =* 525). The union of the two data sets was *n=*530 genes. The relationship between the number of NCCMs per gene and the gene length was determined by the Pearson correlation coefficient.

### Comparison of NCCM Rates between MB and PA.

To compare the NCCM rates in the MB and PA cohorts, the rate for each gene was calculated as described above, and the resulting rate was then subsequently divided by the number of patients in the respective cohort. This normalization of the NCCM rate per patient still resulted in a higher rate of NCCMs in MB than in PA. The NCCM rates of all genes in the genome as well as the top 0.5% of genes in each cohort were compared between MB and PA using unpaired Student’s *t* tests. To test whether the elevated NCCM rates around candidate genes were inflated due to confounders we ran correlation analysis (Pearson correlation) between NCCM rates and local mutation rates as well as the GC content of those regions. The GC contents were calculated with BEDTools (v2.29.2). No strong correlations were found. To determine whether low complexity regions played a role as a confounding factor, we retrieved repeat regions for hg19 from the UCSC table browser (http://genome.ucsc.edu/cgi-bin/hgTables) and intersected them with the positions of NCCMs in the top 0.5% of genes using BEDTools (v2.29.2). NCCMs in the top genes were found in low-complexity regions less often than expected based on the fraction of the whole genome covered by low-complexity regions.

### NCCM Rate Correlation Analysis between MB Subgroups.

The NCCM rate was calculated separately for MB patients of the SHH, group 3 and group 4 subtypes, as well as for pediatric (< 18 y) and adult (≥ 18 y) patients. To determine whether the NCCM rate around specific genes could be used to separate patient subgroups, the subgroup-specific NCCM rates of the 114 candidate genes were further correlated pairwise using the Kendall rank correlation coefficient.

### Evaluation of the Putative Functional Impact of the NCCMs.

To verify whether the NCCMs surrounding the candidate genes have regulatory changes, their coordinates were annotated with regulatory annotations corresponding to the hg19 reference assembly, downloaded from either the UCSC genome browser (Genome Reference Consortium GRCh37 version); or ENCODE portal (v2, source data version: ENCODE Jan 2011), or both. These included, among others, information from tracks of transcription factor binding sites (TFBS), regulatory markers (ORegAnno), transcription start sites, enhancer information, and DNase I hypersensitive sites. The topologically associated domains were visualized with Hi-C data on the 3D genome browser. For the visualization of the *NFIX* region, a TAD reaching from approximately 13.0 Mb to 13.8 Mb using raw tissue Fetal_Brain_CP and Fetal_Brain_GZ at a 10-kb resolution and Cortex_DLPFC at a 40-kb resolution was applied.

The tool sTRAP (49) was used to predict whether the NCCMs were likely to alter the binding affinity of transcription factors to mutated versus wild-type sequences. For every NCCM allele and the corresponding wild-type, 20 *bp* upstream and downstream were used as input sequences. The matrices for the analysis were set to the JASPAR vertebrates database (50), and for the background model, the ‘human promoter’ option was selected. The JASPAR database (http://jaspar.genereg.net) was used to obtain information about transcription factor (TF) binding matrices and motifs of interest. Affinity profiles for the top-ranking matrices with *P*-values ≤ 0.05 were recorded as having significantly more TF binding for the input sequences than what could be expected from a random sequence.

### Mutational Signatures.

MB mutational signatures (COSMIC v3.3) (25, 26) were extracted and assigned to individual mutations using SigProfilerAssignment (v0.0.29) with the export_probabilities_per_mutation parameter set to True (51). Mutational signatures that were assigned to >= 5 % of mutations in at least one sample and were identified in at least 10% of samples were used to compare signature activities across coding mutations, NCCMs, and noncoding nonconstraint mutations (52).

### Pathway Analysis for Candidate Genes.

To gain mechanistic insights into the candidate genes, enrichment analysis was performed using the tool g:GOSt from the online utility g:Profiler (https://biit.cs.ut.ee/gprofiler/gost). First, the options for gene ontology analysis were set to GO- ‘molecular function’, ‘cellular component’, and ‘biological process’. Then, for the pathway analysis, the data sources of KEGG and Reactome pathway databases were selected. Finally, the functional term with enrichment of *P*-values ≤ 0.5, the total number of genes for the term, and the overlap from the query set were tabulated.

### Data Visualization.

To visualize the somatic alterations, oncoprints (brick plots) were generated using tools available on the web-based utility cBioPortal (53). Histograms were plotted using either R or Matlab plotting tools. Custom UCSC genome-browser views were generated by building NCCM tracks for a specified genomic locus, followed by selection of annotation tracks that included TFBS and OregAnno (from ‘Regulation’ track), and ‘Multiz’ alignments of 100 vertebrates. These specific track combinations were saved in a session and reproduced as needed.

### Cell Cultures.

The MB cell line MB002 representing group 3 (54) was a kind gift from Dr. Y.-J.Cho (Oregon Health and Science University, Portland, Oregon, USA) and was cultured in 1:1 mixture of Neurobasal without vitamin A (Life Technologies, Stockholm, Sweden) and DMEM/F12 (Life Technologies) supplemented with 1% nonessential amino acids (Life Technologies), 1 mM sodium pyruvate (Life Technologies), 250 mM Hepes (Life Technologies), 1% glutaMAX, B27 (Life Technologies), heparin (Stemcell Technologies, Grenoble, France), leukemia inhibitory factor (Merck Millipore, Billerica, MA, USA), 20 ng/mL of fibroblast growth factor 2 (FGF2), and 20 ng/mL epidermal growth factor. The MB cell line DAOY representing the SHH Group was grown in Dulbecco’s Modified Eagle Medium supplemented with 10% fetal bovine serum. Both cell lines were maintained with cell culture antibiotics (1× Penicillin-Streptomycin).

### EMSA.

EMSA was run as per the established protocol described previously (53). Briefly, oligos (100 μM) were first annealed in equimolar amounts in 1× annealing buffer (50 mM NaCl, 10 mM Tris-HCl, 10 mM MgCl2, 100 μg/mL BSA, pH 7.9 at 25 °C) in a thermocycler by heating to 95 °C for 5 min and gradual cooling at 1 °C/min to 4 °C. Nuclear proteins were extracted from the MB cell line MB002 using the NucBuster™ Protein Extraction Kit (Millipore). The subsequent binding reaction of the dsDNA with nuclear protein extract was processed using LightShift™ Chemiluminescent EMSA Kit (Thermo Scientific) as per the manufacturer’s protocol, and the binding reaction was conducted by incubation of 8 μg of dsDNA-nuclear protein extract on ice for 40 min. The reaction was stopped by the addition of 5 μL of loading buffer to each binding reaction. A total of 20 μL per reaction was then immediately loaded per well onto a Bio-Rad Criterion gel (Bio-Rad) and electrophoresed for 90 min at 200 V. The gel was transferred to a GeneScreen Plus nylon hybridization transfer membrane (PerkinElmer) for 1 h at 45 V followed by UV crosslinking for 15 min with the membrane facing down on a transilluminator and additional 1 more min with the membrane turned over. The membrane was developed using the Chemiluminescent Nucleic Acid Detection Module Kit (Thermo Scientific) and visualized on the Bio-Rad CCD camera (Bio-Rad). All the sequence probes were designed using the GRCh37/hg19 assembly, and the observed mutations for the NCCMs were inserted in the oligo sequence as required (Dataset S14). All oligos were ordered in sets of three for each wt/NCCM allele pair from Integrated DNA Technologies, with one 5′ biotin-labeled and the same as unlabeled forward strand DNA oligo and one reverse complementary unlabeled strand (HPLC-purified).

### Reporter Assays.

gBlock DNA fragments (IDT) of about 250 bp centered on NCCM and including either reference or mutant allele were cloned into a modified pGL4.26 vector (Promega) with a 9-bp barcode sequence inserted before the poly(A) site. The plasmids were transformed into TOP10 competent cells (Invitrogen), and individual clones were validated by Sanger sequencing. The selected plasmids were pooled together and with a control vector without insert, retransformed, and purified as a pool with EndoFree Plasmid Maxi Kit (Qiagen). Transfection of the cells (MB002) was performed using plasmids pooled together (reporter constructs and control empty vector) using Lipofectamine 3,000 according to the manufacturer’s protocol. Cells were harvested 48 h post transfection in TRIZOL (Thermo Fisher Scientific), and total RNA and DNA were purified according to the manufacturer’s protocol. One μg of RNA/sample was treated with RQ1 RNase-free DNase (Promega) and converted into cDNA using 1 U of M-MuLV reverse transcriptase, oligo(dT) primers, 1 mM dNTPs, and RNase inhibitor (all from Thermo Fisher Scientific). Reporter transcript levels of each allelic construct and empty vector were measured by quantitative real-time PCR using cDNA, and independently, the same fragments were analyzed by qPCR on genomic DNA. The forward primer was common for all plasmids, and reverse primers were specific for plasmid barcodes, generating amplicons of 124 bp that were detected using SYBR green (Thermo Fisher Scientific) by QuantStudio 6 Flex (Applied Biosystems) real-time PCR system. Reporter transcript levels were normalized to the levels of the plasmid DNA using the standard curve method. The experiment was performed three times with five replicates per plasmid pool per experiment. Statistical analyses of all transfections were performed using unpaired Student’s *t* test.

### CRISPR/Cas9 Gene Editing.

CRISPR/Cas9 SNV knock-in cell pools were produced by Synthego (https://www.synthego.com/help/advanced-cell-projects) as per their standardized protocol. In brief, the homology of the sequence ~1 kb of the targeted genomic region was determined for the target cells (DAOY). Multiple chemically modified synthetic single guide RNA (sgRNA) with different PAMs were designed. The donor sequences were selected to be between 115 and 250 bp long. Cells were transfected with ribonucleoprotein (RNPs) complexes consisting of sgRNA and spCas9 protein through nucleofection and knock-in efficiency was analyzed using the free online tool ICE (Inference of CRISPR edits) following Sanger sequencing of the region of interest. The sgRNAs that had the highest knock-in efficiency were chosen for further expansion and cell pools for the various SNVs with the best sgRNA were subsequently used for the experiments. Positive control sgRNA (RELA) was always transfected at the same time and edit efficiency was evaluated. The negative controls (mock transfected with Cas9 protein only) were used as WT or experimental control cells for the downstream experiments.

### Expression and Survival Analysis Using Public Databases.

To compare gene expression levels between normal cerebellum and MB or between the MB subgroups, processed gene expression data were downloaded from the Gene Expression Omnibus database repository. The dataset (GSE124814) used for expression analysis is a batch-normalized resource comprising transcription profiles of 1350 MB samples and 291 normal cerebellum samples (36). All statistical analyses were performed on GraphPad software Prism version 9.1.1 (a commercial proprietary scientific 2D graphing and statistics software, San Diego, CA, USA). The significance of differences in the gene expression between individual subgroups was determined using Student’s unpaired *t* test with Welch’s correction.

The expression dataset (GSE85217) containing 763 MB samples, including 70 WNT, 223 SHH, 144 group 3, and 326 group 4 cases (27), was used for survival analysis. The Kaplan–Meier survival analysis was performed on the R2 (http://r2.amc.nl) genomics platform. Briefly, the dataset samples were divided into two groups based on the gene expression for a given gene. In the order of expression, every increasing expression value is used as a cutoff to create the two groups, and the significance was tested using a log-rank test. The most significant expression cutoff for survival analysis was plotted on the Kaplan–Meier curve based on the log-rank test.

### Expression Analysis of MB Cells.

RNA was extracted from cultured cells using the RNeasy kit from Qiagen. Then, 200 ng of RNA was used for cDNA synthesis using High-Capacity RNA-to-cDNA™ Kit (ThermoFisher). Quantitative PCR was performed using SYBR Green master mix (Applied Biosystems, Foster City, CA, USA) on a StepOnePlus real-time PCR system (Applied Biosystems). Samples were amplified in triplicate and data were analyzed using the ΔΔCT method. All statistical analyses were performed with GraphPad Prism as stated above.

### Proliferation Assay and Drug Treatment of MB Cells.

Cell proliferation was assessed in 96 well format for 0, 24, 48, and 72 h using the CyQuant Direct Cell Proliferation Kit (ThermoFisher). Percentage of proliferation compared to the 0-h reading was calculated and plotted. For treatments, cisplatin (1 μg/mL) and dasatinib (1 to 4 μM) were administered at the beginning of the experiment, and cell numbers were monitored as mentioned above. Prior to treatment experiments, naive DAOY cells were exposed to dasatinib at doses 1, 4, 8, and 10 μM and the growth rate was assessed (*SI Appendix*, Fig. S11*B*). All in vitro experiments were analyzed statistically (students *t* test with Welsch correction) using the GraphPad software Prism.

## Supplementary Material

Appendix 01 (PDF)

Dataset S01 (XLSX)

Dataset S02 (XLSX)

Dataset S03 (XLSX)

Dataset S04 (XLSX)

Dataset S05 (XLSX)

Dataset S06 (XLSX)

Dataset S07 (XLSX)

Dataset S08 (XLSX)

Dataset S09 (XLSX)

Dataset S10 (XLSX)

Dataset S11 (XLSX)

Dataset S12 (XLSX)

Dataset S13 (XLSX)

Dataset S14 (XLSX)

Dataset S15 (DOCX)

## Data Availability

All WGS data used in this project are a part of the ICGC project PBCA-DE. Application for access to these data can be submitted to the ICGC Data Access Compliance Office. All study data are included in the article and/or supporting information.
