# Supplementary material for: Using evolutionary constraint to define novel candidate driver genes in medulloblastoma
Source: Proc Natl Acad Sci U S A. 2023 Aug 7;120(33):e2300984120. doi: 10.1073/pnas.2300984120 (PMC10438395; doi:10.1073/pnas.2300984120)
Supplement: Supplementary file 1 — Appendix 01 (PDF) [file pnas.2300984120.sapp.pdf]

PNAS

Supporting Information for:

Using evolutionary constraint to define novel candidate driver genes in medulloblastoma

Ananya Roy[1]†, Sharadha Sakthikumar[2,3]†, Sergey V. Kozyrev[2], Jessika Nordin[1,2], Raphaela Pensch[2], Suvi Mäkeläinen[2], Mats Pettersson[2], Zoonomia Consortium[4], Elinor K. Karlsson[3,5,6], Kerstin Lindblad-Toh[2,3]‡*, Karin Forsberg-Nilsson[1,7] ‡*

Affiliations:
[1]Department of Immunology, Genetics and Pathology, Science for Life Laboratory; Uppsala University; Uppsala, Sweden.

[2] Department of Medical Biochemistry and Microbiology, Science for Life Laboratory; Uppsala University; Uppsala, Sweden.

[3] Broad Institute; Cambridge, Massachusetts, USA.

[4] Zoonomia Consortium, see supplementary information for collaborators

[5] Program in Molecular Medicine, UMass Chan Medical School; Worcester, MA 01605, USA

[6] Program in Bioinformatics and Integrative Biology, UMass Chan Medical School; Worcester, MA 01605, USA.

[7] Division of Cancer and Stem Cells, University of Nottingham Biodiscovery Institute, Nottingham, UK

†These authors contributed equally to this work
‡ Shared last authors

*Karin Forsberg-Nilsson and Kerstin Lindblad-Toh

Email: karin.nilsson@igp.uu.se and kersli@broadinstitute.org

This PDF file includes:

Figures S1 to S11
Zoonomia Consortium Authorlist

Other supporting materials for this manuscript include the following:

Dataset S1 to S14

SUPPLEMENTARY FIGURES AND LEGENDS

Supplementary Figure 1

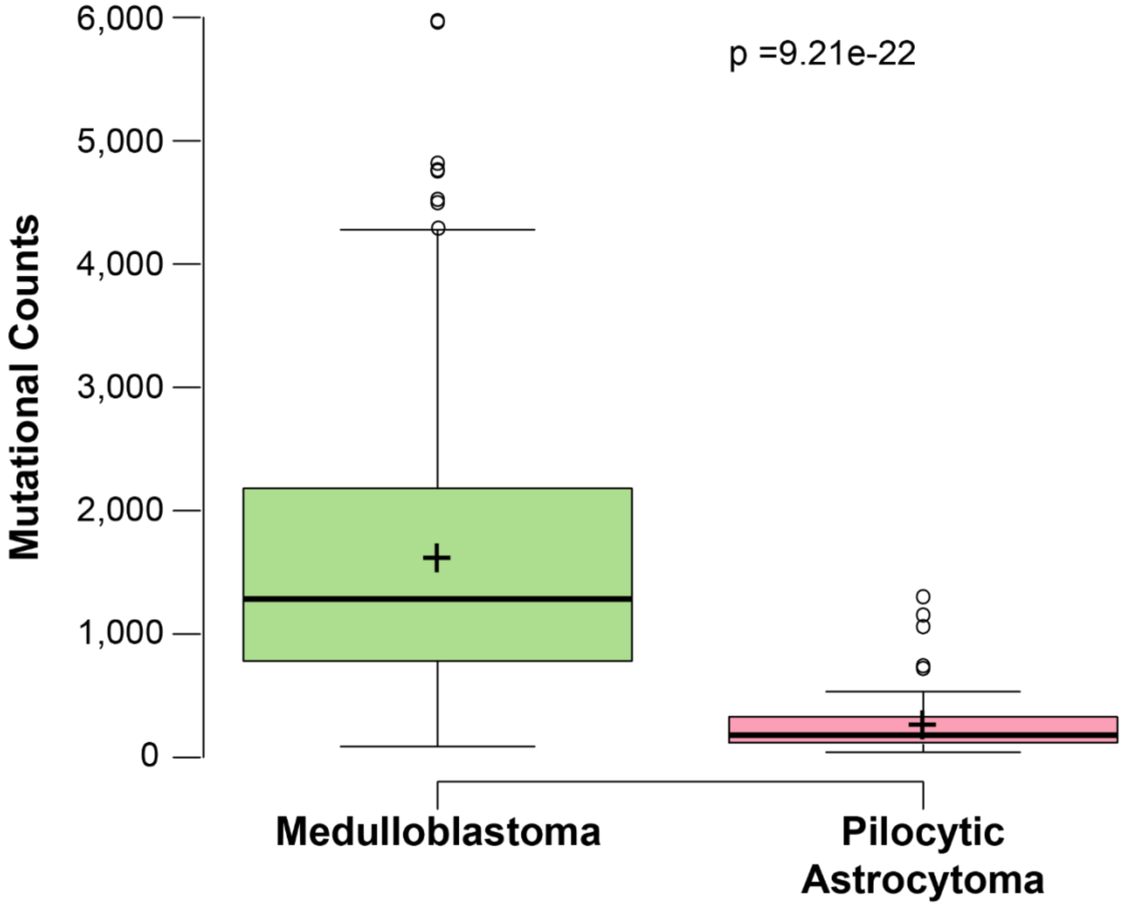

Figure S1: Mutational counts for MB are higher than what is observed for PA. Boxplot depicting the total number of mutations in MB versus PA. Median, the middle data point is represented as a line in the middle of the boxplot and the upper whiskers represent the maximum value within 1.5 * interquartile range of the upper quartile. Plus signs denote average mutational burden per cohort. Un-paired Student t-test was used to analyze the cohorts and the p values for the statistical differences is 9.2e-[22].

Supplementary Figure 2

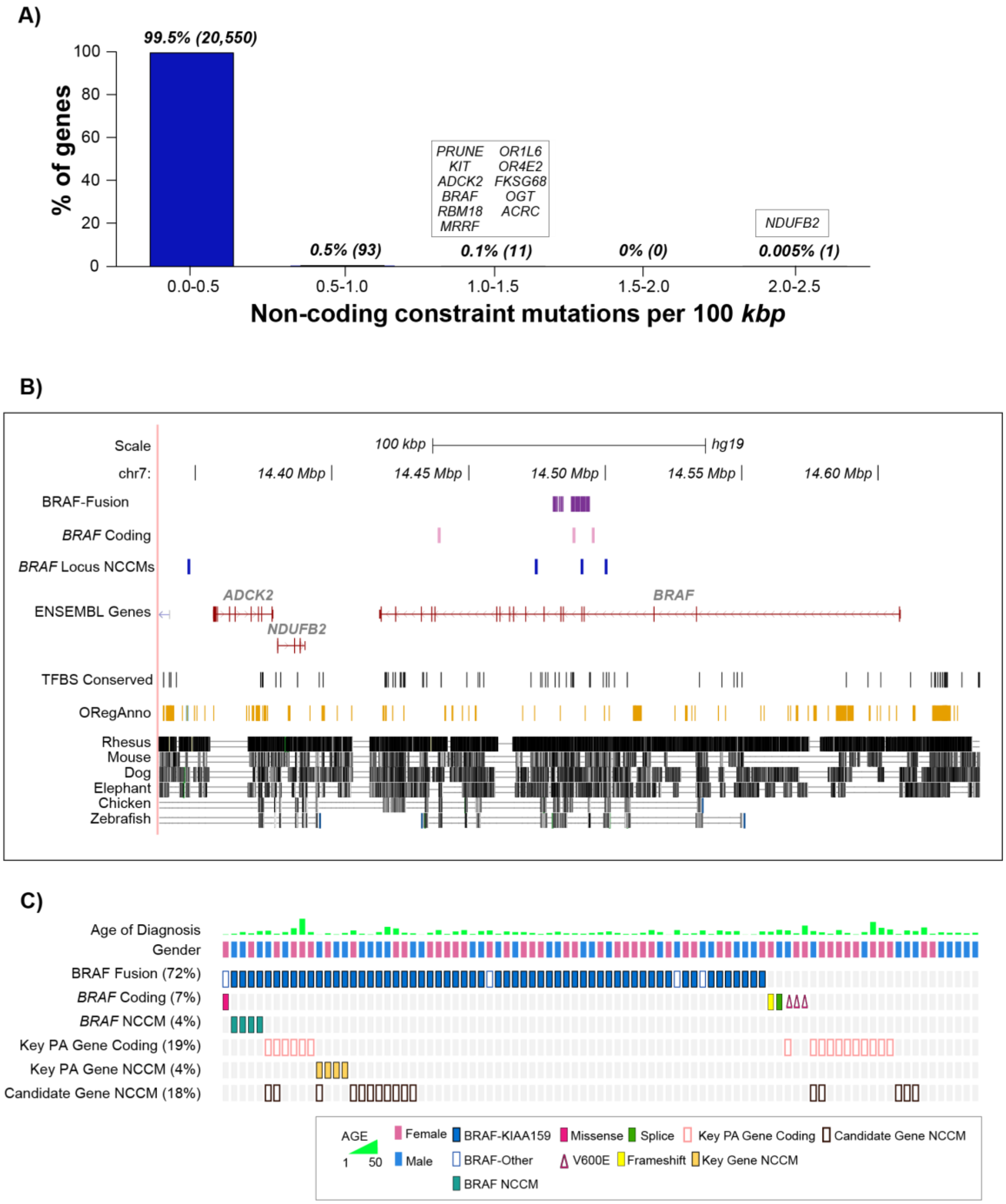

Figure S2: Genes in the *BRAF* locus harbor non-coding constraint mutations that may have driver roles in PA. A) Eleven genes with NCCMs ≥ 1 per 100 kbp were observed, with three of them in the *BRAF* locus. *BRAF* is known to have putative driver functions in PA. B) A UCSC genome browser view shows that the top gene *NDUFB2* shares NCCMs with the genes *BRAF* and *ADCK2*. NCCMs are shown in dark blue, genes in red, transcription factor binding sites in black, and ORegAnno in orange. Lastly, the MultiZ track, which displays a measure of evolutionary

conservation, shows that the *BRAF* locus NCCMs are found in regions of high mammalian conservation. C) Oncoplot shows the somatic changes in the *BRAF* locus and in key protein-coding genes for the PA cohort. (Key genes are genes that have been previously implicated in PA: *BRAF, FGFR1, NF1, KRAS, AHNAK2, AMBP, FLG, IL4R, KIAA1549, PTPN11*, and *SETD2*).

Supplementary Figure 3

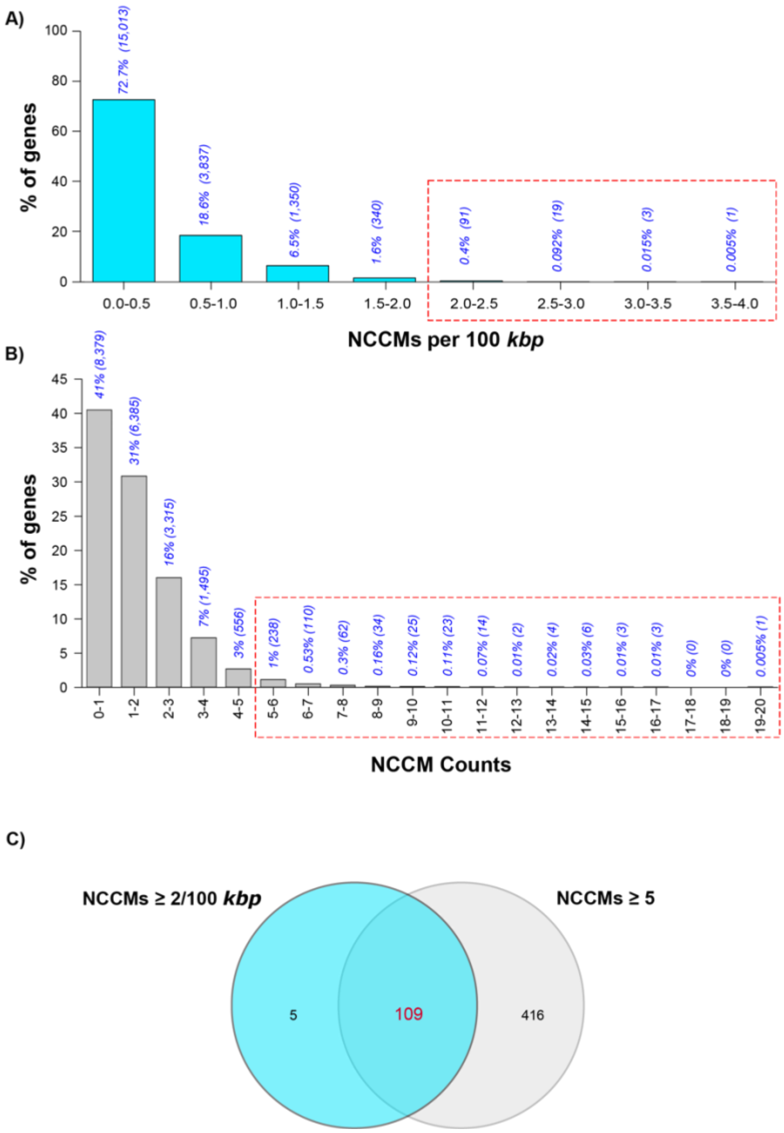

Figure S3: NCCM raw counts versus normalized for length. A) Rates of non-coding constraint mutation for MB genes. Altogether 114 genes had ≥2.0 NCCMs/ 100kbp. B) Number of non-coding constraint mutation per gene in MB samples. Altogether 525 genes had ≥ 5 NCCMs within ±100 kbp. C) Venn diagram showing overlap of data sets in A) and B). The union is 530 genes.

Supplementary Figure 4

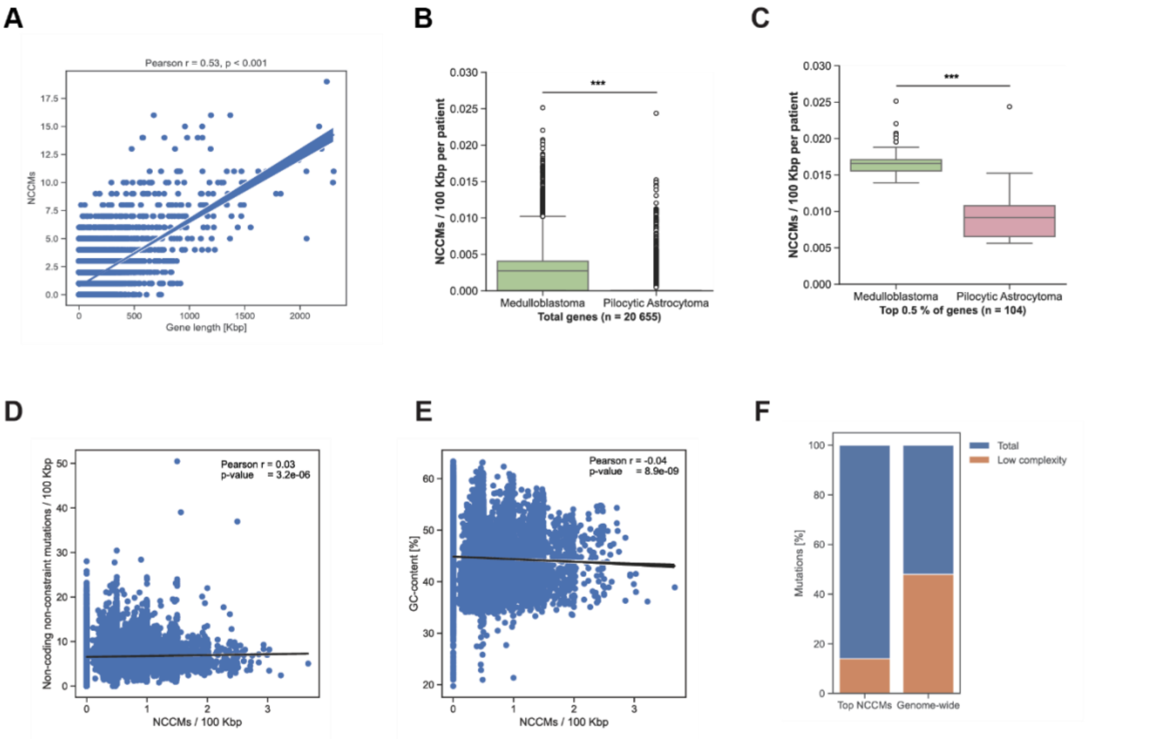

Figure S4: Cohort-wide correlation analysis of NCCMs show that MB had greater NCCM accumulation rates than PA, and that the NCCMs are not a function of confounders. A) Pearson correlation comparing NCCMs per gene to gene length. B) The NCCM rate per patient of all the genes in the genome of MB and PA patients and, C) The top 0.5% of these genes with the highest NCCM rates in each cohort were compared between MB and PA. D-E) Pearson correlation comparing all non-coding non-constraint mutations/100 kbp in MB to all NCCMs/100 kbp per gene (D), and GC content to NCCMs/100 kbp (E). F) Distribution of low complexity regions among NCCMs and genome-wide. Unpaired Student t-test was used to compare NCCM rates between the cohorts (B,C), and the p values for the statistical difference are indicated in the figure. (*** = p ≤ 0.0001).

Supplementary Figure 5

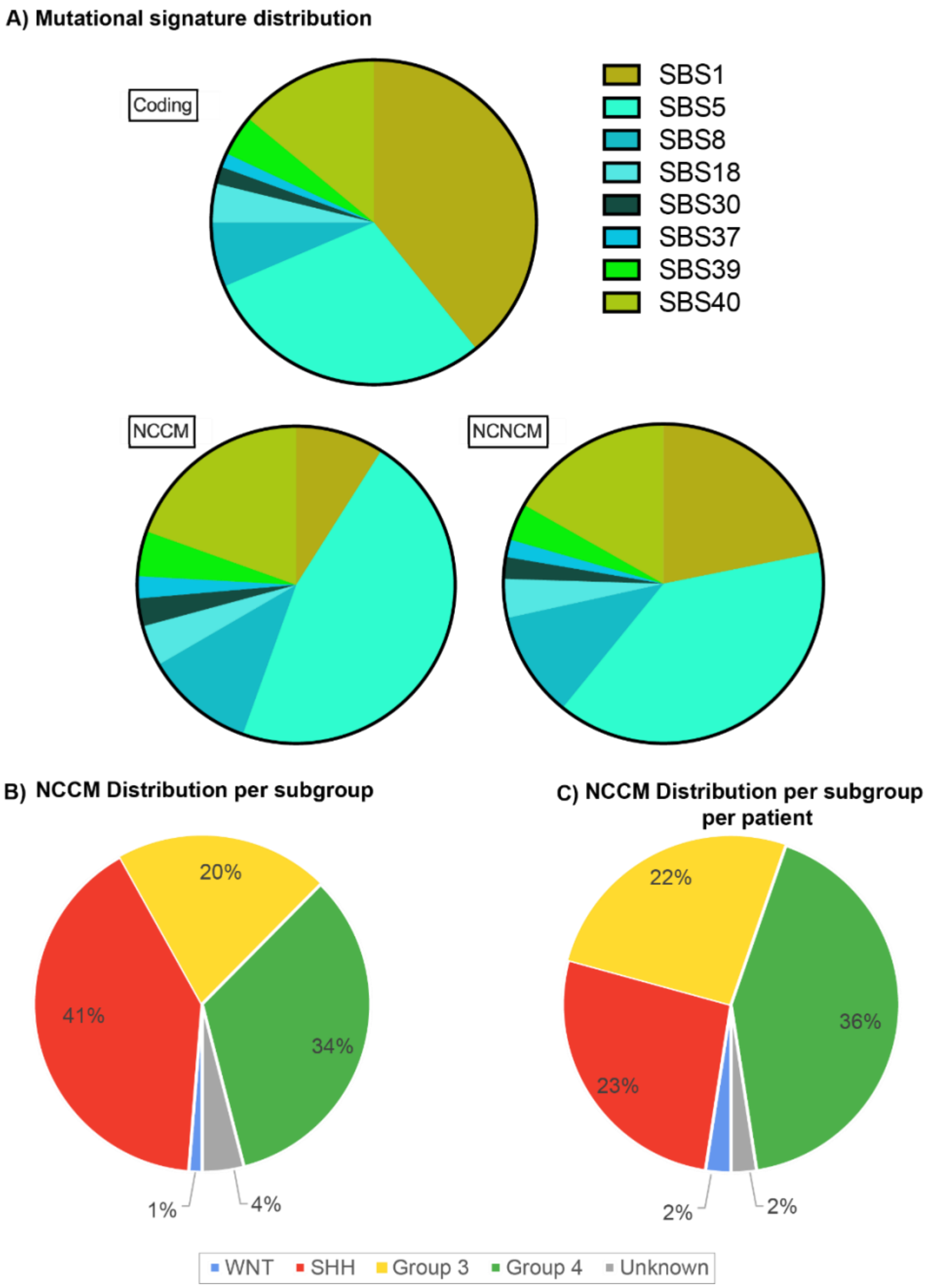

Figure S5: Mutational signatures, and distribution of NCCMs among MB molecular subgroups. A) Pie-chart depicting the distribution of mutational signatures between coding, non-coding non-constraint mutations (NCNCM) and NCCMs. B) Distribution of NCCMs per molecular subgroup. C) Distribution of NCCMs per patient per molecular subgroup.

Supplementary Figure 6

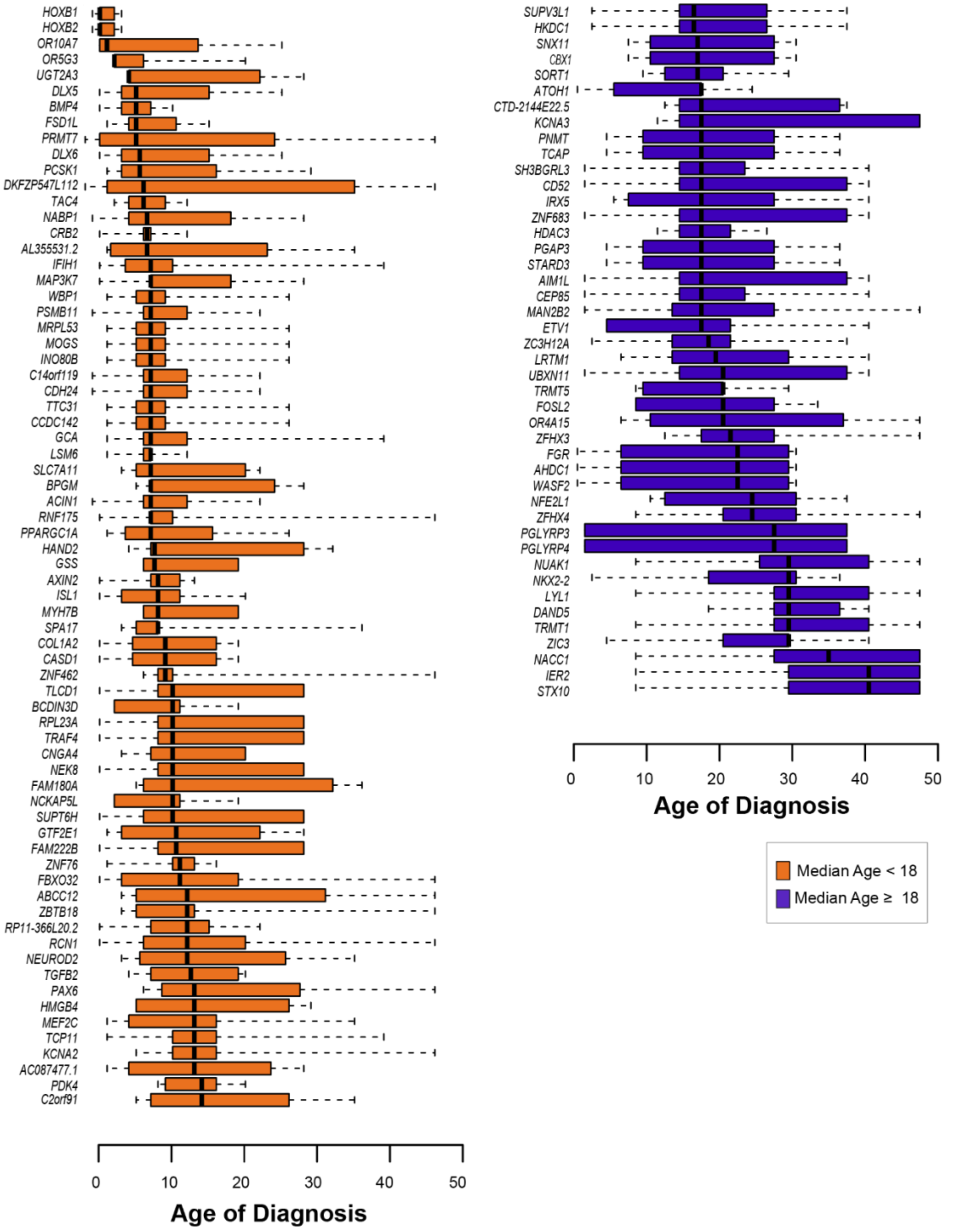

Figure S6: Candidate driver genes for MB (all 114 genes ≥ 2 NCCMs per 100 kbp) are differently distributed across pediatric and adult MB. Orange, median age <18, purple, median age >18.

Supplementary Figure 7

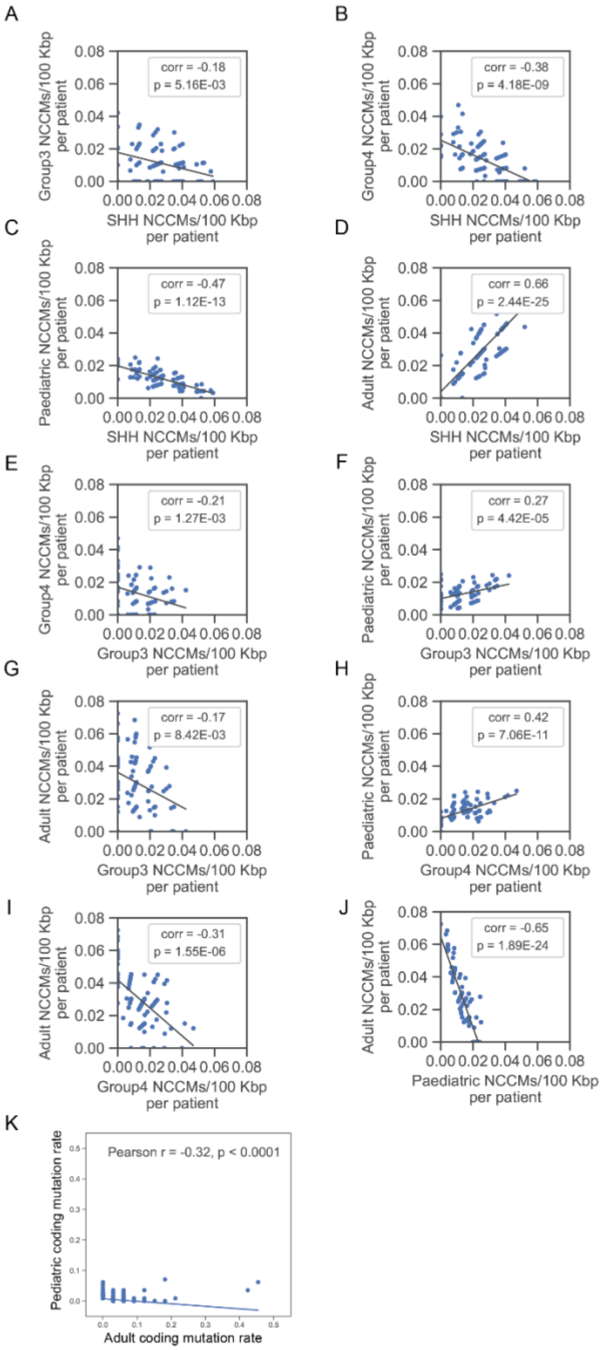

Figure S7: Correlation between NCCM rates in subgroups, and age distribution of patients
A-J) Correlation between NCCMs/100 kbp per patient in genes with ≥2 NCCMs/100 kbp for each combination of subgroups, and age group. Pediatric patients and group 4 were positively correlated (H) while pediatric and adult groups were negatively correlated (J). K) Correlation analysis of coding mutations in pediatric versus adult cohort showed a weak inverse correlation.

Supplementary Figure 8

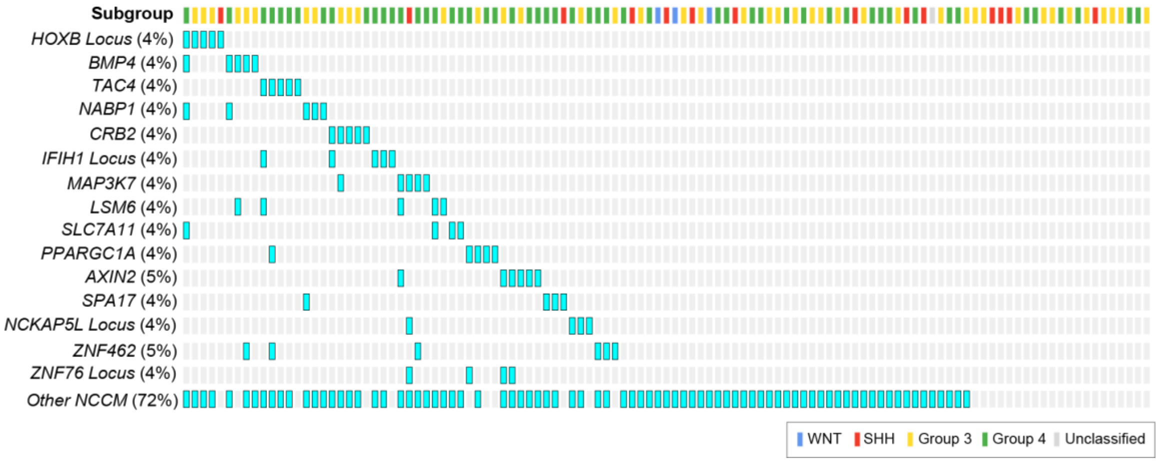

Figure S8: Oncoplots for MB patients
Oncoplot of genes with ≥2 NCCMs/100 kbp that are mainly found in pediatric MB patients. Blue box denotes a patient with NCCM(s).

Supplementary Figure 9

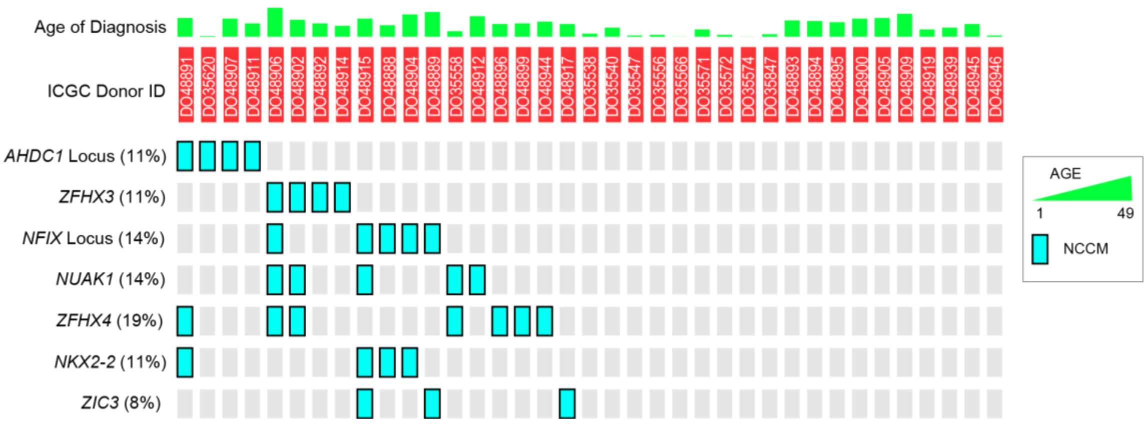

Figure S9: NCCMs predominantly found in adult SHH patients
Oncoplot of somatic changes observed among the seven loci with NCCMs mainly found in adults, ~50% of patients have ≥1 NCCM. Blue box denotes an NCCM.

Supplementary Figure 10

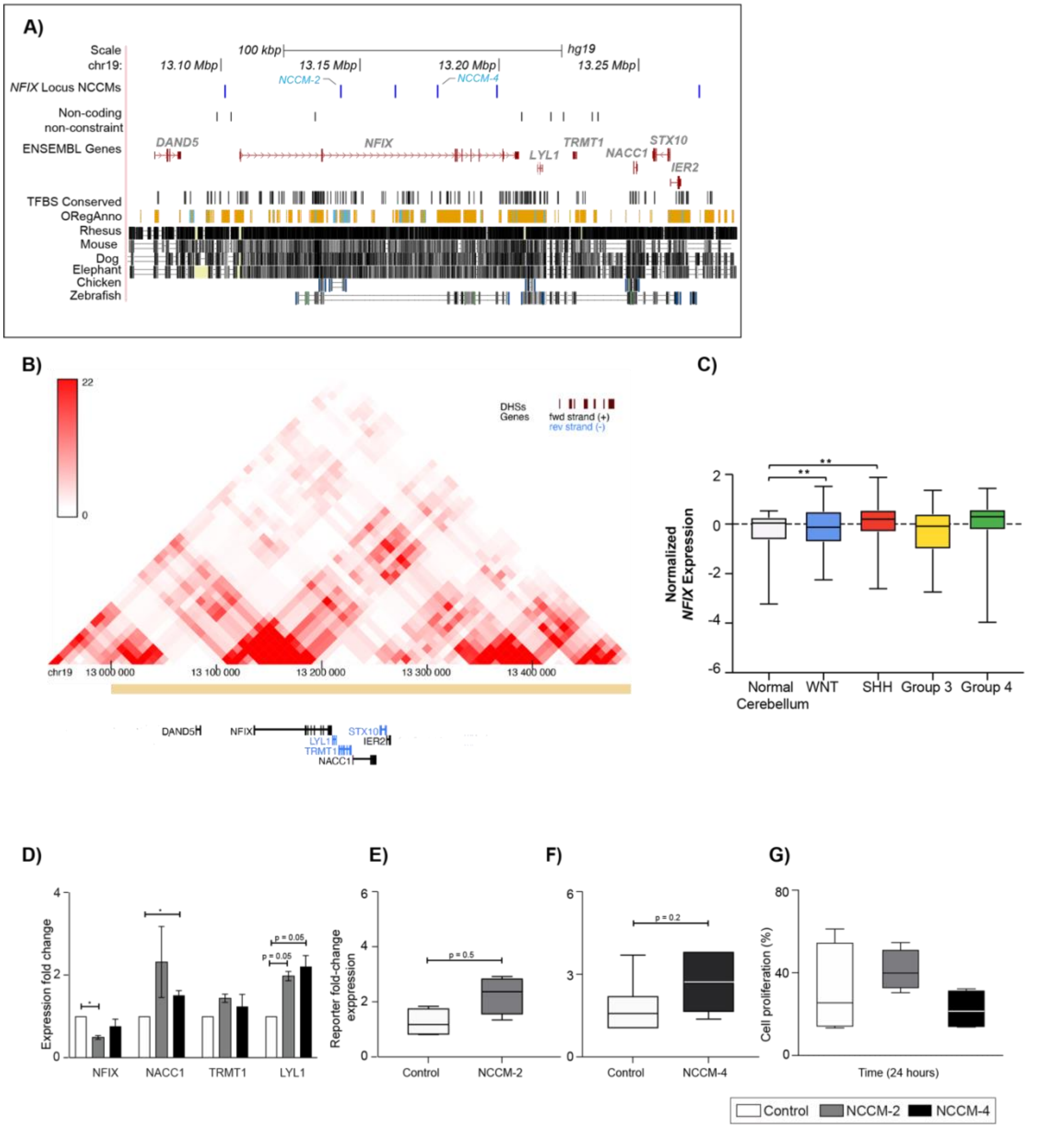

Figure S10: NCCMs in the *NFIX* locus in adult patients

A) In the *NFIX* locus six NCCMs (blue) are shared across seven candidate genes. B) Topologically associated domains for the *NFIX* gene cluster in fetal brain. C) Expression of *NFIX* is higher in WNT and SHH MB subgroups, compared to normal cerebellum. Boxes min-max range, horizontal lines median value, and whiskers extend to extreme values. The significance of differences in the gene expression between individual subgroups was determined using Student's unpaired t-test with Welch's correction. D) CRISPR/Cas9-editing of NCCM-2 and NCCM-4 in DAOY cells results in decreased expression of *NFIX* for NCCM-2, and increased expression of *NACC1* by NCCM-4, while *LYL1* was up-regulated both by NCCM-2 and NCCM-4. E-F) Allelic effects of NCCM-2 € and NCCM-4 (F) on reporter transcript expression did not reach a significant difference (p=0.53, p=0.15) relative to the levels of the wild type reporter vector in the MB002 cells. G) Proliferation assay of edited NCCM-2, NCCM-4 and control DAOY cells did not reach a significant difference. Un-paired

Student t-test with Welch's correction was used to analyze significance. *, ** indicates $p \leq 0.05$ and $p \leq 0.01$ respectively.

Supplementary Figure 11

A

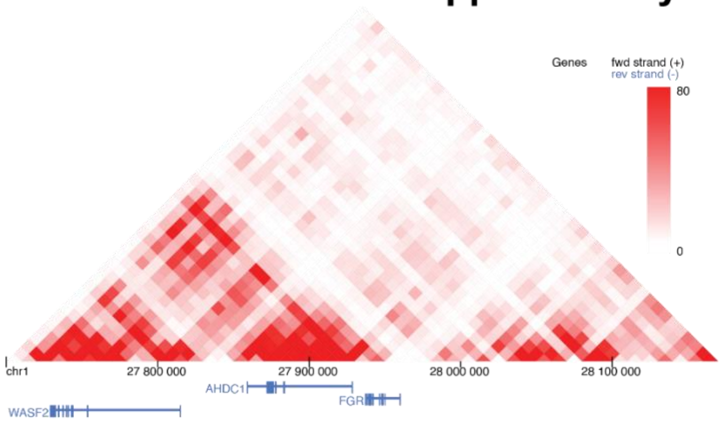

B

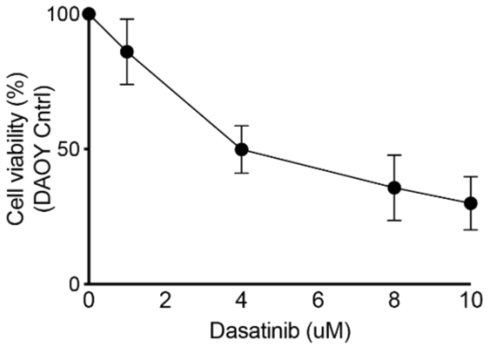

Figure S11: TAD for the *AHDC1* locus and dose reponse test
A) Topologically associated domains for the *AHDC1* gene cluster in fetal brain. B) Dose-response for cell viability of control DAOY cells at 24 hour post treatment with dasatinib.

Zoonomia Authors

The following authors were part of the Zoonomia Consortium:

Gregory Andrews[1], Joel C. Armstrong[2], Matteo Bianchi[3], Bruce W. Birren[4], Kevin R. Bredemeyer[5], Ana M. Breit[6], Matthew J. Christmas[3], Hiram Clawson[2], Joana Damas[7], Federica Di Palma[8,9], Mark Diekhans[2], Michael X. Dong[3], Eduardo Eizirik[10], Kaili Fan[1], Cornelia Fanter[11], Nicole M. Foley[5], Karin Forsberg-Nilsson[12,13], Carlos J. Garcia[14], John Gatesy[15], Steven Gazal[16], Diane P. Genereux[4], Linda Goodman[17], Jenna Grimshaw[14], Michaela K. Halsey[14], Andrew J. Harris[5], Glenn Hickey[18], Michael Hiller[19,20,21], Allyson G. Hindle[11], Robert M. Hubley[22], Graham M. Hughes[23], Jeremy Johnson[4], David Juan[24], Irene M. Kaplow[25,26], Elinor K. Karlsson[1,4,27], Kathleen C. Keough[17,28,29], Bogdan Kirilenko[19,20,21], Klaus-Peter Koepfli[30,31,32], Jennifer M. Korstian[14], Amanda Kowalczyk[25,26], Sergey V. Kozyrev[3], Alyssa J. Lawler[4,26,33], Colleen Lawless[23], Thomas Lehmann[34], Danielle L. Levesque[6], Harris A. Lewin[7,35,36], Xue Li[1,4,37], Abigail Lind[28,29], Kerstin Lindblad-Toh[3,4], Ava Mackay-Smith[38], Voichita D. Marinescu[3], Tomas Marques-Bonet[39,40,41,42], Victor C. Mason[43], Jennifer R. S. Meadows[3], Wynn K. Meyer[44], Jill E. Moore[1], Lucas R. Moreira[1,4], Diana D. Moreno-Santillan[14], Kathleen M. Morrill[1,4,37], Gerard Muntané[24], William J. Murphy[5], Arcadi Navarro[39,41,45,46], Martin Nweeia[47,48,49,50], Sylvia Ortmann[51], Austin Osmanski[14], Benedict Paten[2], Nicole S. Paulat[14], Andreas R. Pfenning[25,26], BaDoi N. Phan[25,26,52], Katherine S. Pollard[28,29,53], Henry E. Pratt[1], David A. Ray[14], Steven K. Reilly[38], Jeb R. Rosen[22], Irina Ruf[54], Louise Ryan[23], Oliver A. Ryder[55,56], Pardis C. Sabeti[4,57,58], Daniel E. Schäffer[25], Aitor Serres[24], Beth Shapiro[59,60], Arian F. A. Smit[22], Mark Springer[61], Chaitanya Srinivasan[25], Cynthia Steiner[55], Jessica M. Storer[22], Kevin A. M. Sullivan[14], Patrick F. Sullivan[62,63], Elisabeth Sundström[3], Megan A. Supple[59], Ross Swofford[4], Joy-El Talbot[64], Emma Teeling[23], Jason Turner-Maier[4], Alejandro Valenzuela[24], Franziska Wagner[65], Ola Wallerman[3], Chao Wang[3], Juehan Wang[16], Zhiping Weng[1], Aryn P. Wilder[55], Morgan E. Wirthlin[25,26,66], James R. Xue[4,57], Xiaomeng Zhang[4,25,26]

[1] Program in Bioinformatics and Integrative Biology, UMass Chan Medical School, Worcester, MA 01605, USA.
[2] Genomics Institute, University of California Santa Cruz, Santa Cruz, CA 95064, USA.
[3] Department of Medical Biochemistry and Microbiology, Science for Life Laboratory, Uppsala University, Uppsala 751 32, Sweden.
[4] Broad Institute of MIT and Harvard, Cambridge, MA 02139, USA.
[5] Veterinary Integrative Biosciences, Texas A&M University, College Station, TX 77843, USA.
[6] School of Biology and Ecology University of Maine, Orono, ME 04469, USA.
[7] The Genome Center, University of California Davis, Davis, CA 95616, USA.
[8] Genome British Columbia, Vancouver, BC, Canada.
[9] School of Biological Sciences, University of East Anglia, Norwich, UK.
[10] School of Health and Life Sciences, Pontifical Catholic University of Rio Grande do Sul, Porto Alegre 90619-900, Brazil.
[11] School of Life Sciences, University of Nevada Las Vegas, Las Vegas, NV 89154, USA.
[12] Biodiscovery Institute, University of Nottingham, Nottingham, UK.
[13] Department of Immunology, Genetics and Pathology, Science for Life Laboratory, Uppsala University, Uppsala 751 85, Sweden.
[14] Department of Biological Sciences, Texas Tech University, Lubbock, TX 79409, USA.
15 Division of Vertebrate Zoology, American Museum of Natural History, New York, NY 10024, USA.

[16] Keck School of Medicine, University of Southern California, Los Angeles, CA 90033, USA. [17] Fauna Bio Incorporated, Emeryville, CA 94608, USA.
[18] Baskin School of Engineering, University of California Santa Cruz, Santa Cruz, CA 95064, USA.
[19] Faculty of Biosciences, Goethe-University, 60438 Frankfurt, Germany.
[20] LOEWE Centre for Translational Biodiversity Genomics, 60325 Frankfurt, Germany.
[21] Senckenberg Research Institute, 60325 Frankfurt, Germany.
[22] Institute for Systems Biology, Seattle, WA 98109, USA.
[23] School of Biology and Environmental Science, University College Dublin, Belfield, Dublin 4, Ireland.
[24] Department of Experimental and Health Sciences, Institute of Evolutionary Biology (UPF-CSIC), Universitat Pompeu Fabra, Barcelona 08003, Spain.
[25] Department of Computational Biology, School of Computer Science, Carnegie Mellon University, Pittsburgh, PA 15213, USA.
[26] Neuroscience Institute, Carnegie Mellon University, Pittsburgh, PA 15213, USA.
[27] Program in Molecular Medicine, UMass Chan Medical School, Worcester, MA 01605, USA.
[28] Department of Epidemiology & Biostatistics, University of California San Francisco, San Francisco, CA 94158, USA.
[29] Gladstone Institutes, San Francisco, CA 94158, USA.
[30] Center for Species Survival, Smithsonian's National Zoo and Conservation Biology Institute, Washington, DC 20008, USA.
[31] Computer Technologies Laboratory, ITMO University, St. Petersburg 197101, Russia.
[32] Smithsonian-Mason School of Conservation, George Mason University, Front Royal, VA 22630, USA.
[33] Department of Biological Sciences, Mellon College of Science, Carnegie Mellon University, Pittsburgh, PA 15213, USA.
[34] Senckenberg Research Institute and Natural History Museum Frankfurt, 60325 Frankfurt am Main, Germany.
[35] Department of Evolution and Ecology, University of California Davis, Davis, CA 95616, USA.
[36] John Muir Institute for the Environment, University of California Davis, Davis, CA 95616, USA.
[37] Morningside Graduate School of Biomedical Sciences, UMass Chan Medical School, Worcester, MA 01605, USA.
[38] Department of Genetics, Yale School of Medicine, New Haven, CT 06510, USA.
[39] Catalan Institution of Research and Advanced Studies (ICREA), Barcelona 08010, Spain. [40] CNAG-CRG, Centre for Genomic Regulation, Barcelona Institute of Science and Technology (BIST), Barcelona 08036, Spain.
[41] Department of Medicine and Life Sciences, Institute of Evolutionary Biology (UPF-CSIC),
Universitat Pompeu Fabra, Barcelona 08003, Spain.
[42] Institut Català de Paleontologia Miquel Crusafont, Universitat Autònoma de Barcelona, 08193 Cerdanyola del Vallès, Barcelona, Spain.
[43] Institute of Cell Biology, University of Bern, 3012 Bern, Switzerland.
[44] Department of Biological Sciences, Lehigh University, Bethlehem, PA 18015, USA.
[45] Barcelona beta Brain Research Center, Pasqual Maragall Foundation, Barcelona 08005, Spain.
[46] CRG, Centre for Genomic Regulation, Barcelona Institute of Science and Technology (BIST), Barcelona 08003, Spain.

[47] Department of Comprehensive Care, School of Dental Medicine, Case Western Reserve University, Cleveland, OH 44106, USA.
[48] Department of Vertebrate Zoology, Canadian Museum of Nature, Ottawa, ON K2P 2R1, Canada.
[49] Department of Vertebrate Zoology, Smithsonian Institution, Washington, DC 20002, USA. [50] Narwhal Genome Initiative, Department of Restorative Dentistry and Biomaterials Sciences, Harvard School of Dental Medicine, Boston, MA 02115, USA.
[51] Department of Evolutionary Ecology, Leibniz Institute for Zoo and Wildlife Research, 10315 Berlin, Germany.
[52] Medical Scientist Training Program, University of Pittsburgh School of Medicine, Pittsburgh, PA 15261, USA.
[53] Chan Zuckerberg Biohub, San Francisco, CA 94158, USA.
[54] Division of Messel Research and Mammalogy, Senckenberg Research Institute and Natural History Museum Frankfurt, 60325 Frankfurt am Main, Germany.
[55] Conservation Genetics, San Diego Zoo Wildlife Alliance, Escondido, CA 92027, USA.
[56] Department of Evolution, Behavior and Ecology, School of Biological Sciences, University of California San Diego, La Jolla, CA 92039, USA.
[57] Department of Organismic and Evolutionary Biology, Harvard University, Cambridge, MA 02138, USA.
[58] Howard Hughes Medical Institute, Chevy Chase, MD, USA.
[59] Department of Ecology and Evolutionary Biology, University of California Santa Cruz, Santa Cruz, CA 95064, USA.
[60] Howard Hughes Medical Institute, University of California Santa Cruz, Santa Cruz, CA 95064, USA.
[61] Department of Evolution, Ecology and Organismal Biology, University of California Riverside, Riverside, CA 92521, USA.
[62] Department of Genetics, University of North Carolina Medical School, Chapel Hill, NC 27599, USA.
[63] Department of Medical Epidemiology and Biostatistics, Karolinska Institutet, Stockholm, Sweden.
[64] Iris Data Solutions, LLC, Orono, ME 04473, USA.
[65] Museum of Zoology, Senckenberg Natural History Collections Dresden, 01109 Dresden, Germany.
[66] Allen Institute for Brain Science, Seattle, WA 98109, USA.
