# Supplementary material for: Using evolutionary constraint to define novel candidate driver genes in medulloblastoma
Source: Proc Natl Acad Sci U S A. 2023 Aug 7;120(33):e2300984120. doi: 10.1073/pnas.2300984120 (PMC10438395; doi:10.1073/pnas.2300984120)
Supplement: Supplementary file 16 — Dataset S15 (DOCX) [file pnas.2300984120.sd15.docx]

**Zoonomia Authors**

The following authors were part of the Zoonomia Consortium:

Gregory Andrews^1^, Joel C. Armstrong^2^, Matteo Bianchi^3^, Bruce W. Birren^4^, Kevin R. Bredemeyer^5^, Ana M. Breit^6^, Matthew J. Christmas^3^, Hiram Clawson^2^, Joana Damas^7^, Federica Di Palma^8,9^, Mark Diekhans^2^, Michael X. Dong^3^, Eduardo Eizirik^10^, Kaili Fan^1^, Cornelia Fanter^11^, Nicole M. Foley^5^, Karin Forsberg-Nilsson^12,13^, Carlos J. Garcia^14^, John Gatesy^15^, Steven Gazal^16^, Diane P. Genereux^4^, Linda Goodman^17^, Jenna Grimshaw^14^, Michaela K. Halsey^14^, Andrew J. Harris^5^, Glenn Hickey^18^, Michael Hiller^19,20,21^, Allyson G. Hindle^11^, Robert M. Hubley^22^, Graham M. Hughes^23^, Jeremy Johnson^4^, David Juan^24^, Irene M. Kaplow^25,26^, Elinor K. Karlsson^1,4,27^, Kathleen C. Keough^17,28,29^, Bogdan Kirilenko^19,20,21^, Klaus-Peter Koepfli^30,31,32^, Jennifer M. Korstian^14^, Amanda Kowalczyk^25,26^, Sergey V. Kozyrev^3^, Alyssa J. Lawler^4,26,33^, Colleen Lawless^23^, Thomas Lehmann^34^, Danielle L. Levesque^6^, Harris A. Lewin^7,35,36^, Xue Li^1,4,37^, Abigail Lind^28,29^, Kerstin Lindblad-Toh^3,4^, Ava Mackay-Smith^38^, Voichita D. Marinescu^3^, Tomas Marques-Bonet^39,40,41,42^, Victor C. Mason^43^, Jennifer R. S. Meadows^3^, Wynn K. Meyer^44^, Jill E. Moore^1^, Lucas R. Moreira^1,4^, Diana D. Moreno-Santillan^14^, Kathleen M. Morrill^1,4,37^, Gerard Muntané^24^, William J. Murphy^5^, Arcadi Navarro^39,41,45,46^, Martin Nweeia^47,48,49,50^, Sylvia Ortmann^51^, Austin Osmanski^14^, Benedict Paten^2^, Nicole S. Paulat^14^, Andreas R. Pfenning^25,26^, BaDoi N. Phan^25,26,52^, Katherine S. Pollard^28,29,53^, Henry E. Pratt^1^, David A. Ray^14^, Steven K. Reilly^38^, Jeb R. Rosen^22^, Irina Ruf^54^, Louise Ryan^23^, Oliver A. Ryder^55,56^, Pardis C. Sabeti^4,57,58^, Daniel E. Schäffer^25^, Aitor Serres^24^, Beth Shapiro^59,60^, Arian F. A. Smit^22^, Mark Springer^61^, Chaitanya Srinivasan^25^, Cynthia Steiner^55^, Jessica M. Storer^22^, Kevin A. M. Sullivan^14^, Patrick F. Sullivan^62,63^, Elisabeth Sundström^3^, Megan A. Supple^59^, Ross Swofford^4^, Joy-El Talbot^64^, Emma Teeling^23^, Jason Turner-Maier^4^, Alejandro Valenzuela^24^, Franziska Wagner^65^, Ola Wallerman^3^, Chao Wang^3^, Juehan Wang^16^, Zhiping Weng^1^, Aryn P. Wilder^55^, Morgan E. Wirthlin^25,26,66^, James R. Xue^4,57^, Xiaomeng Zhang^4,25,26^

^1^ Program in Bioinformatics and Integrative Biology, UMass Chan Medical School, Worcester, MA 01605, USA.

^2^ Genomics Institute, University of California Santa Cruz, Santa Cruz, CA 95064, USA.

^3^ Department of Medical Biochemistry and Microbiology, Science for Life Laboratory, Uppsala University, Uppsala 751 32, Sweden.

^4^ Broad Institute of MIT and Harvard, Cambridge, MA 02139, USA.

^5^ Veterinary Integrative Biosciences, Texas A&M University, College Station, TX 77843, USA.

^6^ School of Biology and Ecology University of Maine, Orono, ME 04469, USA.

^7^ The Genome Center, University of California Davis, Davis, CA 95616, USA.

^8^ Genome British Columbia, Vancouver, BC, Canada.

^9^ School of Biological Sciences, University of East Anglia, Norwich, UK.

^10^ School of Health and Life Sciences, Pontifical Catholic University of Rio Grande do Sul, Porto Alegre 90619-900, Brazil.

^11^ School of Life Sciences, University of Nevada Las Vegas, Las Vegas, NV 89154, USA.

^12^ Biodiscovery Institute, University of Nottingham, Nottingham, UK.

^13^ Department of Immunology, Genetics and Pathology, Science for Life Laboratory, Uppsala University, Uppsala 751 85, Sweden.

^14^ Department of Biological Sciences, Texas Tech University, Lubbock, TX 79409, USA.

15 Division of Vertebrate Zoology, American Museum of Natural History, New York, NY 10024, USA.

^16^ Keck School of Medicine, University of Southern California, Los Angeles, CA 90033, USA. ^17^ Fauna Bio Incorporated, Emeryville, CA 94608, USA.

^18^ Baskin School of Engineering, University of California Santa Cruz, Santa Cruz, CA 95064, USA.

^19^ Faculty of Biosciences, Goethe-University, 60438 Frankfurt, Germany.

^20^ LOEWE Centre for Translational Biodiversity Genomics, 60325 Frankfurt, Germany.

^21^ Senckenberg Research Institute, 60325 Frankfurt, Germany.

^22^ Institute for Systems Biology, Seattle, WA 98109, USA.

^23^ School of Biology and Environmental Science, University College Dublin, Belfield, Dublin 4, Ireland.

^24^ Department of Experimental and Health Sciences, Institute of Evolutionary Biology (UPF-CSIC), Universitat Pompeu Fabra, Barcelona 08003, Spain.

^25^ Department of Computational Biology, School of Computer Science, Carnegie Mellon University, Pittsburgh, PA 15213, USA.

^26^ Neuroscience Institute, Carnegie Mellon University, Pittsburgh, PA 15213, USA.

^27^ Program in Molecular Medicine, UMass Chan Medical School, Worcester, MA 01605, USA.

^28^ Department of Epidemiology & Biostatistics, University of California San Francisco, San Francisco, CA 94158, USA.

^29^ Gladstone Institutes, San Francisco, CA 94158, USA.

^30^ Center for Species Survival, Smithsonian’s National Zoo and Conservation Biology Institute, Washington, DC 20008, USA.

^31^ Computer Technologies Laboratory, ITMO University, St. Petersburg 197101, Russia.

^32^ Smithsonian-Mason School of Conservation, George Mason University, Front Royal, VA 22630, USA.

^33^ Department of Biological Sciences, Mellon College of Science, Carnegie Mellon University, Pittsburgh, PA 15213, USA.

^34^ Senckenberg Research Institute and Natural History Museum Frankfurt, 60325 Frankfurt am Main, Germany.

^35^ Department of Evolution and Ecology, University of California Davis, Davis, CA 95616, USA.

^36^ John Muir Institute for the Environment, University of California Davis, Davis, CA 95616, USA.

^37^ Morningside Graduate School of Biomedical Sciences, UMass Chan Medical School, Worcester, MA 01605, USA.

^38^ Department of Genetics, Yale School of Medicine, New Haven, CT 06510, USA.

^39^ Catalan Institution of Research and Advanced Studies (ICREA), Barcelona 08010, Spain. ^40^ CNAG-CRG, Centre for Genomic Regulation, Barcelona Institute of Science and Technology (BIST), Barcelona 08036, Spain.

^41^ Department of Medicine and Life Sciences, Institute of Evolutionary Biology (UPF-CSIC),

Universitat Pompeu Fabra, Barcelona 08003, Spain.

^42^ Institut Català de Paleontologia Miquel Crusafont, Universitat Autònoma de Barcelona, 08193 Cerdanyola del Vallès, Barcelona, Spain.

^43^ Institute of Cell Biology, University of Bern, 3012 Bern, Switzerland.

^44^ Department of Biological Sciences, Lehigh University, Bethlehem, PA 18015, USA.

^45^ Barcelona beta Brain Research Center, Pasqual Maragall Foundation, Barcelona 08005, Spain.

^46^ CRG, Centre for Genomic Regulation, Barcelona Institute of Science and Technology (BIST), Barcelona 08003, Spain.

^47^ Department of Comprehensive Care, School of Dental Medicine, Case Western Reserve University, Cleveland, OH 44106, USA.

^48^ Department of Vertebrate Zoology, Canadian Museum of Nature, Ottawa, ON K2P 2R1, Canada.

^49^ Department of Vertebrate Zoology, Smithsonian Institution, Washington, DC 20002, USA. ^50^ Narwhal Genome Initiative, Department of Restorative Dentistry and Biomaterials Sciences, Harvard School of Dental Medicine, Boston, MA 02115, USA.

^51^ Department of Evolutionary Ecology, Leibniz Institute for Zoo and Wildlife Research, 10315 Berlin, Germany.

^52^ Medical Scientist Training Program, University of Pittsburgh School of Medicine, Pittsburgh, PA 15261, USA.

^53^ Chan Zuckerberg Biohub, San Francisco, CA 94158, USA.

^54^ Division of Messel Research and Mammalogy, Senckenberg Research Institute and Natural History Museum Frankfurt, 60325 Frankfurt am Main, Germany.

^55^ Conservation Genetics, San Diego Zoo Wildlife Alliance, Escondido, CA 92027, USA.

^56^ Department of Evolution, Behavior and Ecology, School of Biological Sciences, University of California San Diego, La Jolla, CA 92039, USA.

^57^ Department of Organismic and Evolutionary Biology, Harvard University, Cambridge, MA 02138, USA.

^58^ Howard Hughes Medical Institute, Chevy Chase, MD, USA.

^59^ Department of Ecology and Evolutionary Biology, University of California Santa Cruz, Santa Cruz, CA 95064, USA.

^60^ Howard Hughes Medical Institute, University of California Santa Cruz, Santa Cruz, CA 95064, USA.

^61^ Department of Evolution, Ecology and Organismal Biology, University of California Riverside, Riverside, CA 92521, USA.

^62^ Department of Genetics, University of North Carolina Medical School, Chapel Hill, NC 27599, USA.

^63^ Department of Medical Epidemiology and Biostatistics, Karolinska Institutet, Stockholm, Sweden.

^64^ Iris Data Solutions, LLC, Orono, ME 04473, USA.

^65^ Museum of Zoology, Senckenberg Natural History Collections Dresden, 01109 Dresden, Germany.

^66^ Allen Institute for Brain Science, Seattle, WA 98109, USA.
